# Hybrid Liquid Metal Cathode Enables High‐Performance Intrinsically Stretchable OLEDs

**DOI:** 10.1002/adma.202518254

**Published:** 2025-12-28

**Authors:** Wonbeom Lee, Wei Liu, Cheng Zhang, Seungmin Shin, Jaejun Lee, Jaedong Jang, Sanggil Park, Seungbum Hong, Sihong Wang, Himchan Cho

**Affiliations:** ^1^ Department of Materials Science and Engineering Korea Advanced Institute of Science and Technology (KAIST) Daejeon Republic of Korea; ^2^ Pritzker School of Molecular Engineering The University of Chicago Chicago IL USA; ^3^ Institute of Functional Nano & Soft Materials (FUNSOM) Soochow University Suzhou Jiangsu China; ^4^ Graduate School of Semiconductor Technology School of Electrical Engineering Korea Advanced Institute of Science and Technology (KAIST) Daejeon Republic of Korea

**Keywords:** cathode, electromechanical stability, liquid metal, stamping, stretchable OLEDs

## Abstract

Intrinsically stretchable light‐emitting diodes (LEDs) are essential for next‐generation wearable and implantable optoelectronics. However, achieving high‐performance in intrinsically stretchable LEDs remains elusive due to the absence of a stretchable cathode that concurrently ensures efficient electron injection, mechanical compliance, and high optical reflectance. Here, we introduce a hybrid liquid metal—liquid metal particle (Hyb‐LM) cathode, engineered by selective rupture of surface liquid metal particles (LMPs), which facilitates their transformation into a continuous liquid metal (LM) layer. The resulting bilayer structure, comprising a surface LM layer and an underlying LMP layer, exhibits an exceptional combination of low work function (∼4.1 eV), high reflectance (∼90%), low sheet resistance (2.70 × 10^−^^2^ Ω sq^−1^), and negligible resistance changes under 150% strain (R/R_0_ = 1.03 at 150% strain), overcoming fundamental limitations in state‐of‐the‐art stretchable cathodes. The Hyb‐LM cathode enables the realization of intrinsically stretchable organic LEDs with a low turn‐on voltage of 3.0 V, a maximum luminance of 17 670 cd m^−2^, and a record‐high current efficiency of 10.35 cd A^−1^, representing a critical advancement toward stretchable displays and implantable optoelectronics.

## Graphical abstract

We report a hybrid liquid metal (Hyb‐LM) cathode engineered via selective rupture of liquid metal particles (LMPs), forming a bilayer architecture with a surface liquid metal (LM) layer and underlying LMP layer, which has benefits of both LM and LMPs. This cathode simultaneously exhibits low sheet resistance, high reflectance, and low work function, enabling intrinsically stretchable organic light‐emitting diodes with the lowest turn‐on voltage of 3 V, record‐high maximum luminance of 17 670 cd m^−2^, and current efficiency of 10.35 cd A^−1^. The Hyb‐LM design uniquely combines processability, electromechanical robustness, and high‐resolution patternability, advancing stretchable optoelectronics toward next‐generation displays and biomedical integration.

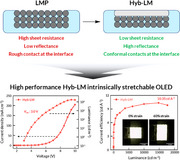

## 1 Introduction

Skin‐like functional electronic devices composed of soft materials are essential for maintaining intimate, imperceptible contact with the complex contours of the human body and organs [1, 2, 3, 4, 5, 6, 7, 8, 9, 10, 11, 12, 13, 14]. Among various functional devices, displays based on light‐emitting diodes (LEDs) [15, 16, 17, 18, 19, 20] represent one of the most critical components, as real‐time visual feedback is a crucial element in human‐machine interfaces. Beyond their use in human‐machine interfaces, LEDs have become key components in diverse areas such as optogenetics [21, 22], phototherapy [23], and biomedical imaging [24]. For these applications, it is necessary to realize displays that combine high stretchability with stable conformal contacts to the human body. Initial efforts to develop stretchable displays primarily focused on utilizing arrays of rigid LEDs connected by stretchable conductors [11, 13, 25, 26] or on fabricating thin organic LEDs (OLEDs) on pre‐stretched elastomeric substrates [27, 28, 29]. While these architectures allowed mechanical deformability to some extent, they suffer from fundamental limitations such as reduced resolution under strain and mechanical incompatibility with soft biological tissues.

To overcome these limitations, efforts have shifted toward the development of intrinsically stretchable LEDs, in which all device components, including the emission layer and electrodes, are inherently stretchable. Various light‐emitting materials, such as fluorescence emitters [30, 31, 32, 33], thermally activated delayed fluorescence (TADF) emitters [34], and colloidal quantum dot (QD) emitters [35], have been explored for this purpose. Nevertheless, high‐performance intrinsically stretchable LEDs have yet to be realized, primarily due to the absence of a cathode that simultaneously ensures efficient electron injection, mechanical compliance, and optical reflectance for single‐sided displays. Conventional stretchable cathodes, including silver nanowire (AgNW) [12, 31, 34], Poly(3,4‐ethylenedioxythiophene) polystyrene sulfonate (PEDOT:PSS) [30], carbon nanotube [36], have high work functions and limited reflectance, thereby impeding efficient electron injection and single‐side light extraction. Liquid metals (LMs), such as Eutectic gallium–indium (EGaIn), offer an attractive option due to their inherently low work (∼4.1 eV) [37], high optical reflectance [38] and mechanical deformability. LMs have been commonly used in the form of liquid metal particles (LMPs) for stretchable electronics due to their negligible resistance change under strain and compatibility with solution‐based patterning process [11, 39, 40]. Nevertheless, their discrete and nonuniform morphology results in poor interfacial contact and significantly reduced reflectance, which limit their applicability in intrinsically stretchable optoelectronics.

**FIGURE 1:**
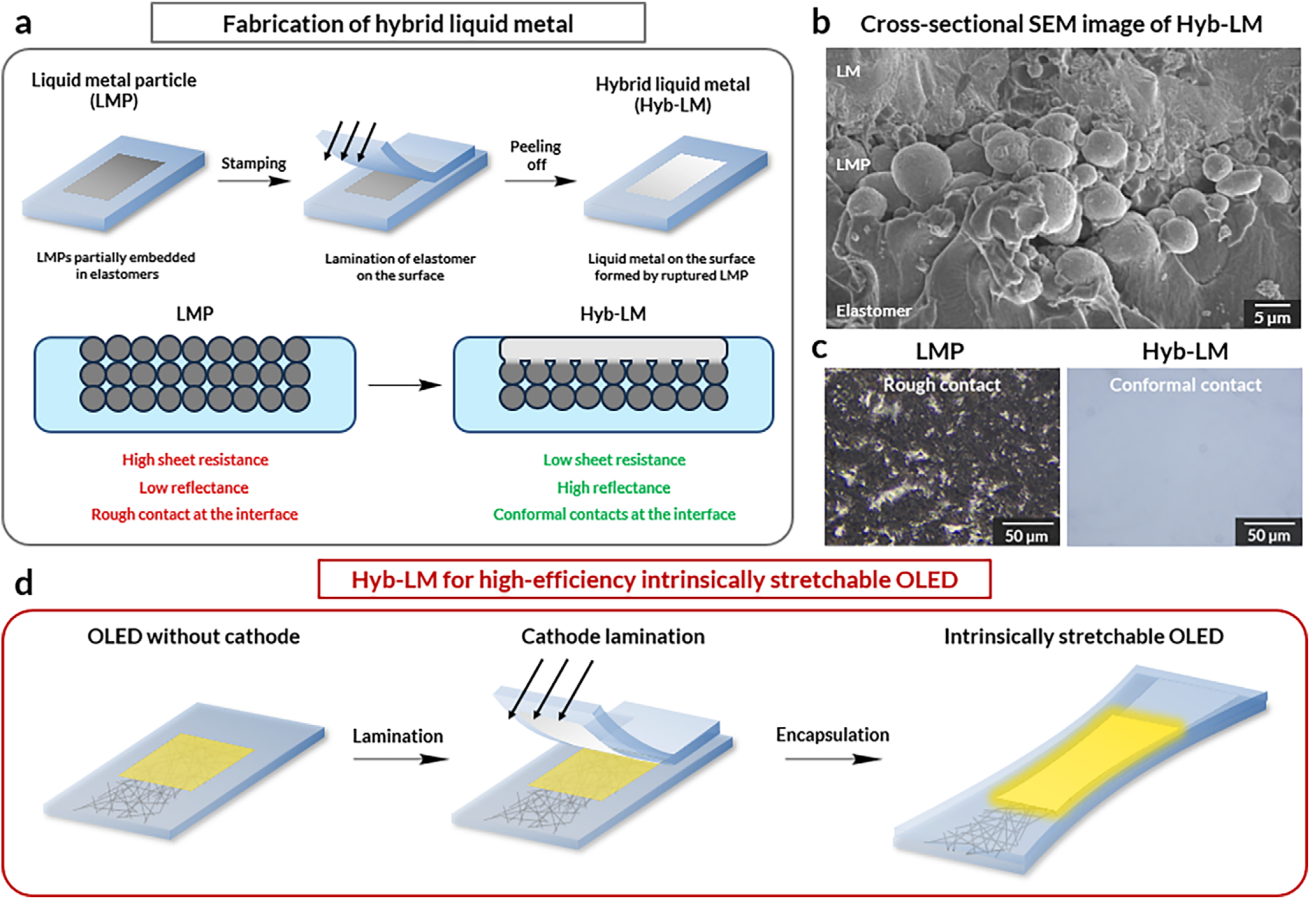
Fabrication of a hybrid liquid metal (Hyb‐LM) cathode and the applications of Hyb‐LM for intrinsically stretchable OLED. (a) Schematic illustration of the fabrication process of Hyb‐LM. (b) Cross‐sectional SEM image of Hyb‐LM, revealing that the surface LM layer is seamlessly connected to the underlying LMP layers. (c) Optical microscope image of LMP and Hyb‐LM laminated on glasses, where the lamination of electrodes on glasses resembles the environment of lamination of the electrodes on spin‐coated layers for LEDs. (d) Schematic illustration of the fabrication of a high‐efficiency intrinsically stretchable OLED using Hyb‐LM.

To address these limitations, EGaIn has been employed in the form of LM via spray‐coating techniques in intrinsically stretchable optoelectronics, including LED [35, 41] and solar cells [42, 43]. While spray‐coated LM films enable conformal contacts in stretchable optoelectronic devices, their practical implementation faces critical challenges: (i) Continuous LM layer formation requires oxidative droplet fusion [35, 41], yet ambient oxygen degrades organic semiconductors, inducing trap states that impede the charge transport and quench electroluminescence via non‐radiative decay. (ii) Limited patternability arising from the high surface tension [44]. (iii) Oxide skins formed during spraying raise an interfacial barrier for electron injection [45]. (iv) Resistance increases under strain [46, 47]. Consequently, no LM design to date meets the electrical, optical, and processability demands for high‐performance intrinsically stretchable LEDs. Here, we present a novel cathode design, termed “Hybrid Liquid Metal (Hyb‐LM)”, which synergistically merges the benefits of LM and LMP with a bilayer architecture (Figure 1a,b; Figure S1). The surface LM layer ensures excellent conductivity and maintains conformal contact with the device (Figure 1c) and maintains its continuous LM layer even at high elongations (Figure S2). The underlying LMP layer serves as a wetting frame, enabling oxide‐free LM patterning under inert conditions (Figure 1b; Figure S3). This synergistic integration delivers unprecedented cathode properties, including an ultralow work function of 2.86 eV with PEIE_PFN‐Br modification (Figure S4), exceptional conductivity (2.70 × 10^−^^2^ Ω sq^−1^), high optical reflectance (∼90%), electromechanical robustness (R/R_0_ = 1.03 at 150% strain), and feasible patterning (200 µm feature). By leveraging the complementary benefits in processibility and materials properties of Hyb‐LM, we successfully achieved high‐performance intrinsically stretchable OLEDs (Figure 1d; Movie S1) with a record‐low turn‐on voltage of 3.0 V, a record‐high luminance of 17 670 cd m^−2^, and a high maximum current efficiency of 10.35 cd A^−1^.

## 2 Fabrication of Hybrid Liquid Metal Electrode

The fabrication of the Hyb‐LM cathode comprises the following two major steps. Initially, a colloidal LMP solution was prepared using a probe sonicator and LMP‐PDMS composites, where LMPs are partially embedded in PDMS, were prepared. Subsequently, a PDMS is utilized to rupture the LMPs at the surface. (See Experimental section and Figure S5 for detailed process). The sonication time for the LMP solution plays a critical role in both the preparation of the LMP‐PDMS composite and the fabrication process. We first examined the morphology of spray‐coated LMPs with the varying sonication time of 5, 10, and 20 s (Figure S6). LMPs spray‐coated with 10 and 20 s of sonication produce the particle sizes of ∼3 and ∼1.5 µm, respectively (Figures S6a,b and S7). However, LMPs spray‐coated with 5 s of sonication couldn't maintain their particulate form and transformed into LM during the spray‐coating process due to the air pressure during the spray‐coating process (Figure S6c). Therefore, we concluded that LMPs with 5 s of sonication cannot be used for LMP‐PDMS composite. We further investigated the effect of the size of particles on the process. Both LMP‐PDMS composites with ∼3 and ∼1.5 µm LMPs have particulate upon peeling off from the wafer. However, after stamping, the surface of electrodes with ∼3 µm LMPs transformed into LM (Figures S8 and S9), whereas electrodes with ∼1.5 µm LMPs retained their particulate form (Figure S10). This behavior is attributed to the influence of the particle size on the mechanical properties of LMPs. As particle size decreases, surface tension and the rigidity of the oxide layer begin to dominate, imparting solid‐like characteristics to the LMPs. Conversely, larger particles exhibit more liquid‐like behavior, as the liquid nature of the LM core prevails (Figure S11) [11]. The size‐dependent mechanical response of LMPs was confirmed by AFM nanoindentation measurements (Figure S12). LMPs with diameters of 1, 3, and 6 µm exhibited elastic moduli of 1.92 GPa, 21.35 MPa, and 3.84 MPa, respectively, showing a substantial decrease in stiffness with increasing particle size. We infer that LMPs sonicated for 5 s were sufficiently large to revert to LM under the pressure exerted during spray‐coating. This was further confirmed by the agitation of 5 s‐sonicated LMP solutions, which led to their transformation into LM (Figure S13), demonstrating their inherent instability. In contrast, 1.5 µm LMPs were too small to rupture even after stamping due to their higher effective Young's modulus, a consequence of their reduced size. This indicates that 3 µm LMPs possess an optimized effective Young's modulus, allowing them to remain in particulate form upon peeling off from the wafer while transforming into LM upon stamping Therefore, we conclude that 3 µm LMPs represent the optimal condition for our methodology.

## 3 Patterning and Materials Properties of Hyb‐LM

**FIGURE 2:**
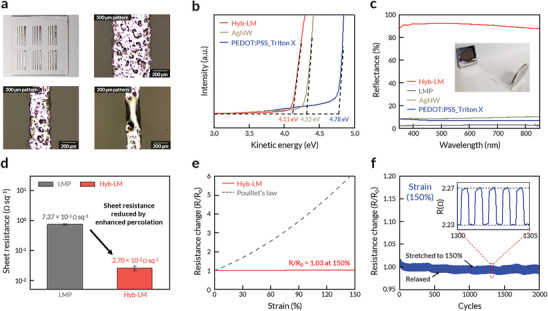
Feasible patterning and material properties of Hyb‐LM for optoelectronics. (a) Optical microscope images of patterned Hyb‐LM, showing the feasible patternability of the Hyb‐LM down to 200 µm. (b) UPS spectra of Hyb‐LM (4.11 eV, red) and conventional stretchable electrodes: AgNW (4.32 eV, olive‐gray), PEDOT:PSS_Triton X (4.78 eV, dark blue). (c) Reflectance of Hyb‐LM (red), LMP (gray), AgNW (olive‐gray) and PEDOT:PSS_Triton X (dark blue) as a function of the wavelength from 350 to 850 nm. Inset is a photo of reflected image of a one cent coin on Hyb‐LM, which further confirms the high reflectivity of Hyb‐LM. (d) Sheet resistance of LMP and Hyb‐LM. The sheet resistance decreased from 7.37 × 10^−1^ ± 3.7 × 10^−2^ Ω sq^−1^ (LMP) to 2.70 × 10^−2^ ± 4.3 × 10^−3^ Ω sq^−1^ (Hyb‐LM), due to the enhanced percolation by stamping process. Sheet resistance data are represented as mean values ± s.d. from four 4 samples. (e) Relative resistance changes of Hyb‐LM under uniaxial strain up to 150% (red line) and the theoretical resistance change predicted by Pouillet's law (gray dot, R/R_0_ = (1+ ε)^2^, where ε is the applied strain). (f) Cyclic stability of Hyb‐LM under 150% strain up to 2000 cycles. Inset shows a detailed response of the resistance of Hyb‐LM to the applied strain.

Patterning of pristine LM is crucial for the development of intrinsically stretchable LEDs. However, achieving this has been a significant challenge due to the fluid nature and high surface tension of LM. Our method, however, enables high‐precision electrode patterning with a resolution feature down to 200 µm, facilitated by a simple solution process (Figure 2a; Figures S14 and S15). Hyb‐LM with various shapes (e.g., star, heart, and dot; Figure S16) was further demonstrated, confirming the versatility of our method. We investigated the characteristics of Hyb‐LM as a stretchable cathode material. The work function is a critical parameter in determining suitability for electron injection. Ultraviolet photoelectron spectroscopy (UPS) measurements revealed that Hyb‐LM exhibits a work function of approximately 4.11 eV, similar to that of EGaIn [37] and comparable to aluminum — the most widely used cathode material in rigid LEDs. In contrast, conventional stretchable electrodes such as AgNW and PEDOT:PSS_Triton X have higher work functions of 4.32 and 4.78 eV, respectively, making them inefficient for electron injection from the cathode to the emission layer (EML) (Figure 2b; Figure S17). To achieve high light‐out coupling efficiency in stretchable optoelectronic devices, a device architecture incorporating a transparent electrode coupled with a highly reflective counter electrode is essential. We evaluated the reflectance of electrodes by placing them on the quartz substrate. As shown in Figure 2c, Hyb‐LM exhibited high reflectance (∼90%), whereas AgNW, PEDOT:PSS_Triton X, and LMP showed low reflectance (<10%). The high reflectance of Hyb‐LM is attributed to its inherently reflective nature and conformal contact. In contrast, the low reflectance of AgNW and PEDOT:PSS can be attributed to their semi‐transparent nature, while the low reflectance of LMP is likely due to the interfacial light scattering induced by its nonconformal contact with the quartz substrate.

We also measured the sheet resistance of both LMP and Hyb‐LM electrodes, observing a substantial reduction from 7.37 × 10^−1^  Ω sq^−1^ for the LMP electrode to 2.70 × 10^−^^2^ Ω sq^−1^ for the Hyb‐LM electrode (Figure 2d). The percolation pathways in the LMP electrode rely on dot‐to‐dot contact between particles, which leads to limited charge transport. In contrast, the surface of the Hyb‐LM electrode consists of a continuous LM layer, forming an effective conducting path that significantly lowers the sheet resistance (Figure S18).

The Hyb‐LM electrode also demonstrated excellent electromechanical properties, exhibiting negligible resistance change at 150% strain (R/R_0_ = 1.03) (Figure 2e). It maintained its electromechanical performance over 2000 stretching cycles at strains of 50%, 100%, and 150% (Figure 2f; Figure S19). Furthermore, the Hyb‐LM retained its electrical stability under various deformation modes, including bending and twisting, further confirming its excellent mechanical robustness and durability under repeated mechanical stress (Figure S20). Notably, the electromechanical properties of the Hyb‐LM electrode surpass those of previously reported LM electrodes (Table S1) [11, 42, 47, 48, 49, 50, 51, 52]. The resistance of the Hyb‐LM electrode is influenced by both LM and underlying LMP layers. While the resistance of LM may increase under stretching, the LMP layer remains stable, as the contact area between LMPs increases under strain, effectively compensating for resistance fluctuations (Figure S21) [46, 48].

We further investigated the effect of strain rate on the electromechanical performance of the Hyb‐LM electrode. The Hyb‐LM electrode showed stable electrical performance during dynamic stretching, regardless of strain rate (Figure S22). Furthermore, the electromechanical performance of the Hyb‐LM electrode was superior to that of conventional stretchable electrodes, such as AgNW and PEDOT:PSS_Triton X, which are commonly used as stretchable cathodes in intrinsically stretchable LEDs. (Figure S23).

To sum up, the Hyb‐LM electrode satisfies all essential criteria for a stretchable cathode, including a low work function for efficient electron injection, high reflectance for optimal light extraction, low sheet resistance, and excellent electromechanical properties. The combination of LM at the surface and the underlying LMP layer ensures both high conductivity and mechanical durability, making Hyb‐LM a strong candidate for stretchable optoelectronic devices.

## 4 Hyb‐LM for High‐Performance OLEDs

**FIGURE 3:**
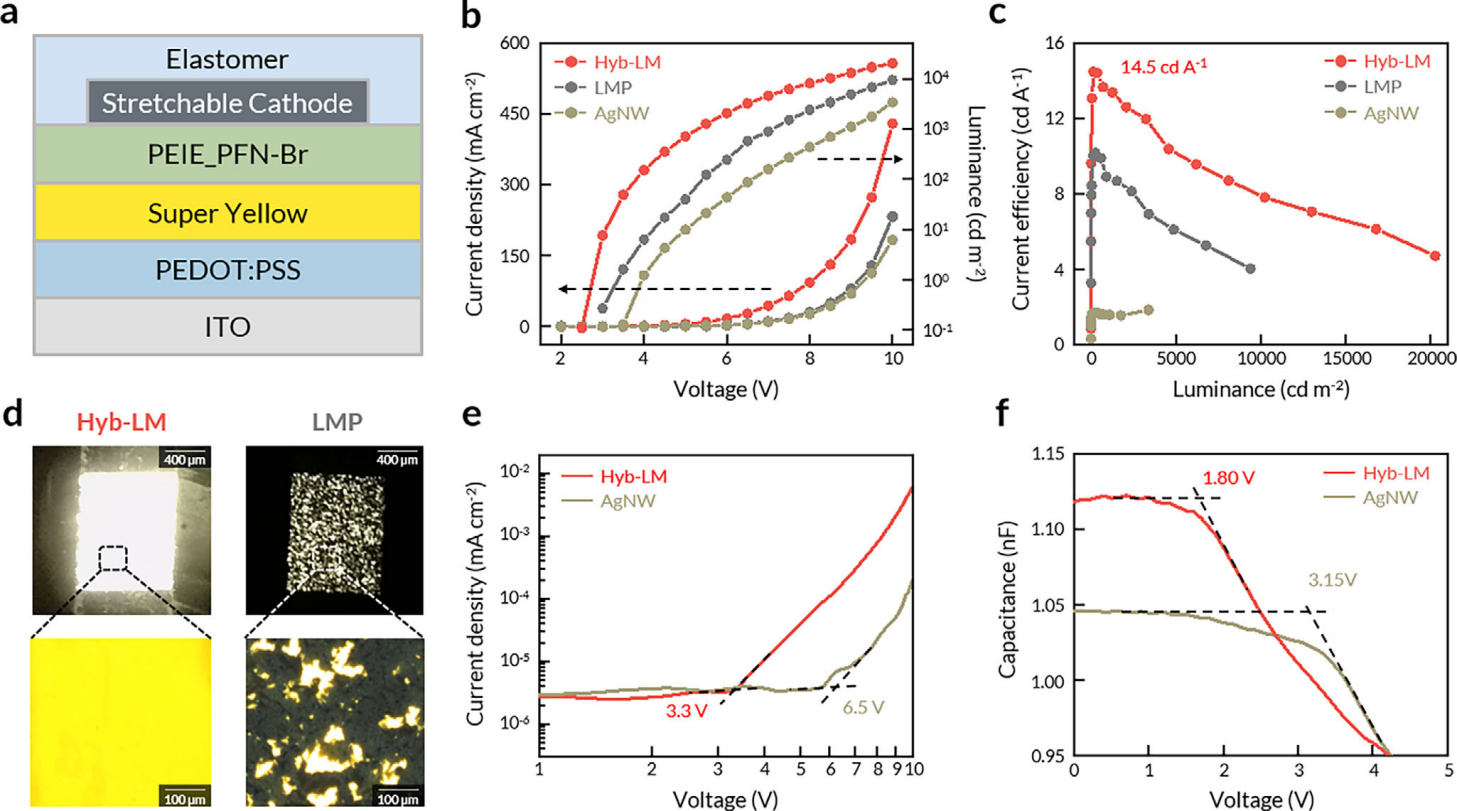
EL performance of rigid OLED using Hyb‐LM cathode. (a) Schematic showing the rigid OLED structure for characterizing EL performances of different stretchable cathodes. (b,c), Representative current density‐luminance‐voltage traces (b) and current efficiency‐luminance traces (c) of SY‐based OLEDs with Hyb‐LM, LMP, and AgNW cathodes, showing that the OLED with Hyb‐LM cathode outperforms OLEDs with LMP and AgNW cathodes in efficiency, turn‐on voltage, and luminance. (d) Photographs (top) and optical microscope (bottom) images of the OLED pixels with Hyb‐LM (left) and LMP (right) at 4 V. (e) Current density‐voltage traces in electron‐only devices. Charge injections were observed at 3.3 V for the Hyb‐LM electrode and 6.5 V for the AgNW electrode. The structure of electron‐only devices: ITO/PEIE_PFN‐Br/SY/PEIE_PFN‐Br/Cathode (Hyb‐LM or AgNW). (f) Capacitance‐voltage traces in OLED with Hyb‐LM and AgNW cathodes. Charge injections were observed at 1.80 V for the Hyb‐LM electrode and 3.15 V for the AgNW electrode.

To assess the impact of Hyb‐LM as a stretchable cathode on OLED efficiency, we investigated the performance of OLEDs incorporating Hyb‐LM and that of reported stretchable cathodes, including LMP and AgNW. OLEDs with a device structure of ITO/PEDOT:PSS/Super Yellow (SY)/Polyethyleneimine ethoxylated: Poly(9,9‐bis(3'‐(N,N‐dimethyl)‐N‐ethylammoinium‐propyl‐2,7‐fluorene)‐alt‐2,7‐(9,9‐dioctylfluorene))dibromide (PEIE_PFN‐Br)/stretchable cathode (Hyb‐LM, LMP, or AgNW) were fabricated (Figure 3a; Figure S24). PEIE_PFN‐Br was chosen as an electron injection layer (EIL) for its high stretchability [34] (Figure S25) and its ability to substantially reduce the work function of the cathode electrode (Figure S26) [53, 54]. Additionally, incorporation of PFN‐Br ensured efficient electron injection [54], making it well‐suited for stretchable EILs. The selection of materials also accounted for energy level alignment to optimize device efficiency (Figures S27 and S28). As illustrated in Figure 3b,c, OLEDs incorporating the Hyb‐LM cathode demonstrated a low turn‐on voltage (2.5 V), high maximum luminance (20 200 cd m^−2^), and high maximum current efficiency (14.5 cd A^−1^). Notably, the efficiency of the Hyb‐LM OLED cathode was comparable to the OLED incorporating a conventional aluminum cathode (Figure S29), highlighting the potential of Hyb‐LM as an excellent cathode for OLEDs. In contrast, OLEDs utilizing LMP and AgNW cathodes showed inferior OLED performances, including higher turn‐on voltages, lower luminance, and lower current efficiencies (Figure 3b). Further comparison with OLEDs incorporating LM cathodes deposited under ambient conditions underscores the advantages of the Hyb‐LM approach (Figure S30). As shown in Figure S30a, Hyb‐LM OLEDs exhibited a significantly higher average current efficiency of 10.56 cd A^−1^ across 15 devices, compared to 0.63 cd A^−1^ for OLEDs using ambient‐processed LM cathodes. This performance disparity is attributed to the degradation of electronic materials under ambient conditions, leading to the formation of trap states and subsequent non‐radiative recombination (Figure S30b).

Such superior device performances in OLEDs with Hyb‐LM cathodes compared to conventional stretchable cathodes were attributed to their low work function (Figure 2b), low sheet resistance (Figure 2d), and conformal interfacial contact with the EIL (Figure 1c). The uniform electroluminescence observed in the Hyb‐LM OLED pixel was enabled by the conformal contact between the EIL and the upper LM layer of the Hyb‐LM cathode, whereas the LMP OLED exhibited significant nonuniformity due to their dot‐like interfacial contact (Figure 3d). To investigate the influence of low work function and sheet resistance on electron injection, we fabricated electron‐only devices (EODs) with the structure of ITO/EIL/EML/EIL/electrode, where PEIE_PFN‐Br and SY served as the EIL and EML, respectively (Figure 3e). As expected, the *J*–*V* characteristics of the EOD with the Hyb‐LM cathode exhibited a substantially lower onset voltage for electron injection (3.3 V) compared to the AgNW‐based EOD (6.5 V) (Figure 3e). This improvement was attributed to a smaller energy difference between the work function of Hyb‐LM and the lowest unoccupied molecular orbital (LUMO) level of the EIL (Figure S23) and the low sheet resistance of Hyb‐LM. Capacitance‐voltage (C‐V) measurements provided additional evidence for enhanced electron injection in Hyb‐LM OLEDs. Compared to AgNW OLEDs, the C‐V curve of Hyb‐LM OLEDs exhibited its shoulder peak at a much lower bias (∼1.80 V for Hyb‐LM OLEDs vs. ∼3.15 V for AgNW OLEDs). indicating improved energy level alignment in Hyb‐LM OLEDs (Figure 3f).

To demonstrate the broad applicability of our Hyb‐LM electrode to various types of LEDs, the Hyb‐LM was also incorporated into an indium phosphide (InP) quantum dot LEDs (QLEDs) as the cathode (Figures S31 and S32). Benefiting from the low work function and sheet resistance, QLEDs with Hyb‐LM exhibited efficient charge injection, achieving a turn‐on voltage of 3.0 V and a high luminance of 11 100 cd m^−2^ (Figure S31b).

## 5 High‐Performance Intrinsically Stretchable OLEDs Using Hyb‐LM

**FIGURE 4:**
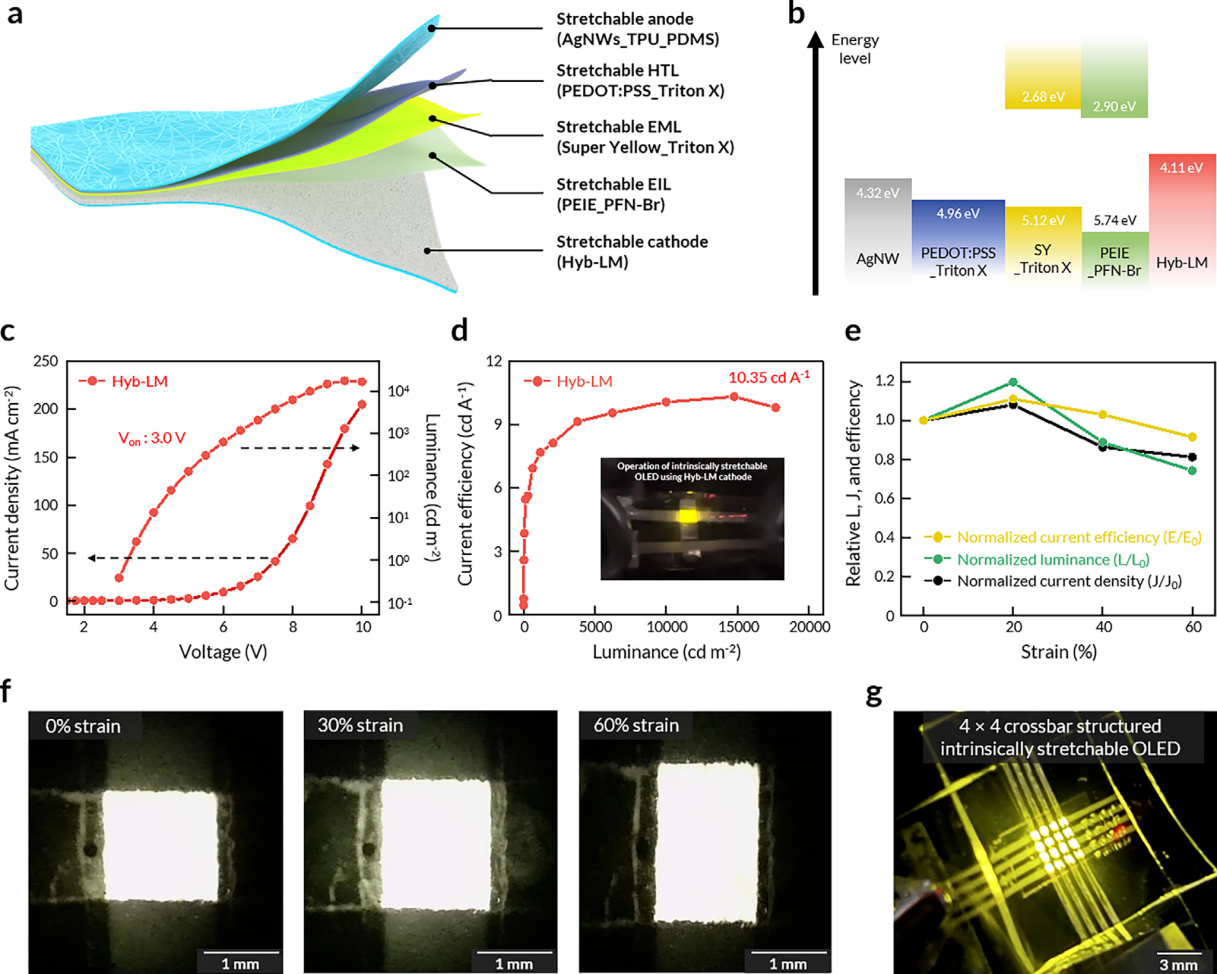
High‐performance intrinsically stretchable OLED using Hyb‐LM. (a) Schematic showing the device structure of the intrinsically stretchable OLED. (b) Energy‐level diagram of the intrinsically stretchable OLED. (c) Representative current density‐luminance‐voltage traces and current efficiency‐luminance trace of the intrinsically stretchable OLED. (d) Current efficiency of the intrinsically stretchable OLED depending on luminance. (e) Relative change of current density, luminance, and current efficiency of the intrinsically stretchable OLED at different strains up to 60%. (f) Photographs of the Hyb‐LM‐based intrinsically stretchable OLED at 0%, 30%, and 60% strain. (g) Photograph of a 4 × 4 crossbar structured intrinsically stretchable OLED.

Based on the results from rigid OLEDs, we utilized our stretchable Hyb‐LM electrode to realize high‐performance intrinsically stretchable OLEDs. The device structure (Figure 4a; Figures S33 and S34) closely resembles that of SY‐based rigid OLEDs, with a major change of the anode from ITO to AgNW embedded in thermoplastic polyurethane (TPU)_PDMS matrix [55]. The materials were selected with consideration for both stretchability and optimal energy level alignment for charge injection, facilitating the realization of high‐performance intrinsically stretchable OLEDs (Figure 4b; Figures S35–S38). Triton X was incorporated into both SY and PEDOT:PSS to improve their stretchability (Figures S39 and S40) [31, 32]. The intrinsically stretchable OLED with Hyb‐LM cathode exhibited a low turn‐on voltage of 3.0 V, high maximum luminance of 17 670 cd m^−2^ and current efficiency of 10.35 cd A^−1^ (corresponding to external quantum efficiency of 3.55% under Lambertian assumption) (Figure 4c,d), while maintaining the stable emission from SY at high operation voltages (Figures S41 and S42). Our device exhibited the lowest turn‐on voltage, highest luminance, and highest current efficiency among reported intrinsically stretchable LEDs, underscoring the exceptional performance of Hyb‐LM as a stretchable cathode (Figure S43 and Table S2) [30, 31, 32, 34, 35, 56, 57, 58, 59]. The slightly lower efficiency and luminance as well as the higher driving voltage in intrinsically stretchable OLEDs compared to rigid SY‐based OLEDs may be attributed to the lower hole injection, resulting from the less effective interfacial contact, lower transparency, and conductance of AgNW compared to ITO [31, 34].

The intrinsically stretchable OLED with Hyb‐LM cathode showed robust operational stability. The device exhibited a half‐lifetime of at 7.41 h at 100 cd m^−2^ and 2.66 h at 600 cd m^−2^ (Figure S44). This performance represents a substantial improvement over previous reports (Table S3) [31, 32, 35, 59]. Under mechanical deformation, the current efficiency, current density, and luminance remained 91%, 81%, and 74% of their initial values, respectively, at unidirectional 60% strain (Figure 4e). Furthermore, the current efficiency remained at 87% of its original value after it was 100 repeated stretching cycles at 40% strain (Figure S45). Notably, our device showed better mechanical‐luminance stability (L/L_0_ = 0.74 at 60% strain) than that of devices based on AgNW cathode (L/L_0_ = 0.60 at 60% strain) [12, 31], which is attributed to the superior electromechanical property of Hyb‐LM compared to AgNW (Figure S23). The observed decrease in EL performance under strain was primarily attributed to the increased resistance of the AgNW electrode and interfacial degradation in the intrinsically stretchable OLEDs. The intrinsically stretchable OLED with Hyb‐LM exhibited a maximum strain of 60%, beyond which microcrack formation in the SY_Triton film occurred, generating leakage current pathways between the Hyb‐LM and AgNW electrodes and leading to device failure (Figure S46). Photographs of the emitted pixel (Figure 4f) and the stable emission color spectra under strains (Figure S47) further revealed that the intrinsically stretchable OLED can be stretched up to 60% without notable degradation. Moreover, we expanded the device architecture to demonstrate an intrinsically stretchable 4 ×  4 crossbar structured intrinsically stretchable OLEDs featuring high‐resolution 500 µm pixels (Figure 4g; Figure S48). Notably, this represents the highest pixel resolution reported among crossbar‐structured intrinsically stretchable LEDs, enabled by the high‐resolution patternability of Hyb‐LM (Table S4) [30, 32, 34, 35].

## 6 Discussion

In this work, we have developed a hybrid liquid metal electrode that combines an oxide‐free LM surface layer with an underlying LMP layer, thereby unifying their advantages, including low sheet resistance (2.70 × 10^−2^ Ω sq^−1^), low work function with PEIE_PFN‐Br (∼2.86 eV), high reflectance (∼90%), excellent electromechanical behavior without losing its electrical conductance up to 150% strain (R/R_0_ = 1.03 under 150% strain), and a patterning resolution down to 200 µm. This bilayer design overcomes the fundamental limitations of conventional stretchable cathodes, including conductance change under strain, low reflectance, and inadequate electron injection. When incorporated into rigid OLEDS, the Hyb‐LM cathode enabled device performance that not only outperformed those using conventional stretchable cathodes but also matched that of OLEDs employing thermally evaporated metal cathodes, demonstrating the suitability of Hyb‐LM as a cathode. Furthermore, integration of Hyb‐LM into intrinsically stretchable OLEDs yielded a device with a high current efficiency (10.35 cd A^−1^), a high luminance (17 670 cd m^−2^), and a low driving voltage (3.0 V). These devices also exhibited stable device performances under 60% strain (E/E_0_ = 0.91, J/J_0_ = 0.81, and L/L_0_ = 0.74), and 40% cyclic strain (E/E_0_ = 0.87). The successful fabrication of high‐resolution, crossbar‐arrayed stretchable OLEDs demonstrates the versatility and scalability of this approach. We envision that the Hyb‐LM electrode will provide a universal platform for next‐generation intrinsically stretchable optoelectronic devices, including those designed for human‐interactive and implantable applications.

## 7 Methods

### 7.1 Materials

Unless otherwise specified, the chemicals and solvents used in the current work were purchased from Sigma–Aldrich and used without further purification. Eutectic Gallium Indium was purchased from Changsha Ruichi Nonferrous Metals. Sylgard 184 was purchased from Dow Corning. PEDOT:PSS (PH1000, AI4083) were purchased from Heraeus. Silver nanowire solution (AW045) was purchased from Zhejiang Kechuang Advanced Materials. Thermoplastic Polyurethane (Elastollan 1185A) was received from BASF. PFN‐Br (Poly[(9,9‐bis(3'‐((N,N‐dimethyl)‐N‐ethylammonium)‐propyl)‐2,7‐fluorene)‐alt‐2,7‐(9,9‐dioctylfluorene)]dibromide), TFB (Poly [(9,9‐dioctylfluorenyl ‐2,7‐diyl)‐co‐(4,4'‐(N‐(4‐sec‐butylphenyl)diphenylamine)]) was purchased from Luminescence Technology Corp. Indium Phosphide quantum dot was purchased from UniAM. Patterned ITO was purchased from AMG.

### 7.2 Surface Treatment for the Substrates

Trichloro (1H,1H,2H,2H‐perfluorooctyl) silane (FDTS) functionalized substrates were prepared using the following methods. Silicon wafers were treated by oxygen plasma (100 W, 50 kHZ) for 5 min. Then the wafer was treated with FDTS vapor for 30 min at 80°C. The substrates were washed with isopropyl alcohol (IPA) after the vapor treatment and stored in ambient conditions. Octadecyltrimethoxysilane (OTS)‐functionalized substrates were prepared using the following methods. Silicon wafers were treated by oxygen plasma (100 W, 50 kHZ) for 5 min, and OTS solution in trichloroethylene (20 µL OTS in 20 mL trichloroethylene) was spin‐coated on the substrate at 0 rpm for 30 s and 3000 rpm for 30 s. The substrates were then treated with ammonium hydroxide vapor in the glass desiccator overnight. The substrates were washed with toluene after the vapor treatment and stored in ambient conditions.

### 7.3 Preparation of the Hyb‐LM Electrode

Eutectic Gallium Indium (EGaIn) microparticles solution was first prepared in IPA. To prepare the solution, 0.5 g EGaIn was injected into 10 mL IPA in a glass vial. An acoustic wave was then applied with 40% amplitude for 10 s using a probe sonicator with the probe diameter of 12 mm (VCX‐500, Sonics and Materials Inc.). Prepared EGaIn microparticles solution was spray‐coated using a spray‐coater having a nozzle diameter of 300 µm, with a gas pressure of 1 bar on the FDTS surface‐treated silicon wafer covered with polyimide tape mask. The solution was shaken right before spray‐coating. The polyimide tape mask was removed after spray‐coating, then Sylgard 184 (15:1 base to crosslinker weight ratio) was drop‐casted on the wafer and fully cured at 80°C for 2 h. The composite was then carefully peeled off from the wafer and stamped with Sylgard 184 (15:1 base to crosslinker weight ratio) in the N_2_‐filled glovebox. Insulating EGaIn microparticles beneath EGaIn at the surface were further electrically activated by applying mechanical force via stretching. The detailed procedure is shown in Figure S5.

### 7.4 Preparation of Silver Nanowire Electrodes

AgNW solution (AW045) was first vortexed for 10 min to remove aggregates, then diluted in IPA (1:19 volume ratio), followed by 10 s bath sonication. Next, the diluted solution was spray‐coated on OTS surface‐treated silicon wafer covered with Kapton tape mask, until the sheet resistance of 3 Ω sq^−1^ was reached. After removing the mask, the AgNW spray‐coated on the wafer was washed with DI water, then dried at 120°C for 10 min. Next, TPU solution (20 mg mL^−1^ in tetrahydrofuran) was spin‐coated on AgNW for 30 s at 3000 rpm, followed by drying at 120°C for 20 min. Dried AgNW covered with TPU were treated with O_2_ plasma for 30 s (100 W, 50 kHz). Then sylgard 184 (15:1 base to crosslinker weight ratio) was drop‐casted onto the TPU layer. The composite was stored in the air overnight, then annealed at 80°C for an hour to be cured. After the PDMS was fully cured, AgNW_TPU_PDMS was carefully peeled off from the wafer to be used. The detailed procedure is illustrated in Figure S33.

### 7.5 Preparation of PEDOT:PSS (PH1000)_Triton X Electrodes

PEDOT:PSS (PH1000) was mixed with 5 wt.% of Triton X, serving as a surfactant and stirred using magnetic stirrer for 3 h. Next, the prepared PEDOT:PSS solution was spin‐coated on the PDMS (15:1 base to crosslinker weight ratio). PDMS was treated with O_2_ plasma for 1 min (100 W, 50 kHz) before spin‐coating.

### 7.6 Measurement of Materials Properties

Sheet resistance was measured using four collinear, equally spaced probes connected to a Keithley 2400 sourcemeter. For electromechanical characterization, rectangular samples (10 mm × 1 mm) were used. Samples were first attached to an electric motor with double‐sided tape, and samples were then connected to external copper wires with EGaIn to minimize the contact resistance at the interface. The pads were encapsulated with scotch tape again for the fixation of the copper wire. The real‐time resistance of Hyb‐LM during the operation of the electric motor was then measured using a Keithley 2400 sourcemeter, which was connected to an external copper wire. Reflectance of materials were measured using a JASCO V‐770 spectrophotometer. The work function of electrodes was measured by UPS measurement using Kratos AXIS Nova. Photoluminescence intensity of SY film was measured using a light‐source‐extendable visible‐to‐NIR spectrofluorometer (Horiba; NFEC‐2024‐12‐302228)

### 7.7 Fabrication and Measurement of Rigid Super Yellow (SY) based OLED

The ITO‐coated glasses were cleaned with 1 vol.% Hellmanex solution, IPA, and deionized water, and then dried and further treated with oxygen plasma (100 W, 50 kHZ) for 5 min. PEDOT:PSS (AI4083) was first spin‐coated on ITO substrates at 4500 rpm for 45 s, followed by annealing at 150°C for 30 min. SY solution (5 mg mL^−1^ in toluene) was spin‐coated on PEDOT:PSS layer at 2000 rpm for 60 s and annealed at 100°C for 30 min. PEIE_PFN:Br (0.25 wt.% in methanol, respectively) was spin‐coated on EML at 3000 rpm for 30 s and annealed at 100°C for 10 min. Prepared electrodes were then gently laminated on top of PEIE_PFN:Br layer. J‐V‐L measurements were carried out using a Keithley 2400 sourcemeter and a spectroradiometer (CS‐2000, Konika Minolta).

### 7.8 Fabrication of Rigid InP QLED

The ITO‐coated glasses were cleaned with 1 vol.% Hellmanex solution, IPA, and deionized water, and then dried and further treated with oxygen plasma (100 W, 50 kHZ) for 5 min. PEDOT:PSS (AI4083) was first spin‐coated on ITO substrates at 4500 rpm for 45 s, followed by annealing at 150°C for 30 min. TFB (8 mg mL^−1^ in chlorobenzene) was spin‐coated on the PEDOT:PSS layer at 3000 rpm for 60 s and annealed at 150°C for 10 min. Then InP solution (20 mg mL^−1^ in Octane) was spin‐coated on the TFB layer at 2000 rpm for 30 s, followed by annealing at 150°C for 10 min. ZnMgO solution was spin‐coated on InP layer at 3000 rpm for 20 s, then annealed at 150°C for 30 min. Prepared electrodes were then gently laminated on top of the ZnMgO layer.

### 7.9 Synthesis of ZnMgO Nanoparticles

ZnMgO used for ZnMgO nanoparticles. Zinc acetate dihydrate (ZnAc, 0.5765 g) and magnesium acetate pentahydrate (MgAc, 0.0805 g) were dissolved in 30 mL of DMSO with vigorous stirring. Tetraethylammonium hydroxide (TMAH) (0.9062 g) was dissolved in 10 mL of ethanol. After 30 min, the TMAH solution was injected rapidly into the ZnAc and MgAc solution. After the solution became transparent, the ZnMgO solution was washed with ethyl acetate, centrifuged for 5 min at 4500 rpm, and redispersed in ethanol.

### 7.10 Fabrication of Intrinsically Stretchable OLED

AgNW_TPU_PDMS electrode was first treated with oxygen plasma for 1 min (100 W, 50 kHz). Then PEDOT:PSS (AI4083) mixed with Triton X (5 wt.%) was spin‐coated at 4500 rpm for 45 s on the AgNW electrode, followed by annealing at 150°C for 30 min. Then SY_Triton X (5, 2.5 mg mL^−1^ respectively in toluene) was spin‐coated on PEDOT:PSS_Triton X at 2000 rpm for 1 min, then annealed at 100°C for 30 min. PEIE_PFN‐Br (0.25 wt.% in MeOH, respectively) was further spin‐coated on SY_Triton X layer at 3000 rpm for 30 s, followed by annealing at 100°C for 10 min. Lastly, the prepared Hyb‐LM was gently laminated on the PEIE_PFN‐Br. Detailed fabrication process is illustrated in Figure S34.

## Author Contributions

W.L., W.L., S.W., and H.C. conceived and designed the experiments and wrote the manuscript. W.L. prepared and characterized Hyb‐LM and other stretchable conductors. W.L. and J.J. fabricated the rigid and intrinsically stretchable OLEDs and performed characterization and measurements. W.L. and C.Z. helped with the fabrication of rigid LEDs and intrinsically stretchable LEDs. W.L. and S.S. performed UPS measurement. J.L. performed XPS measurements. S.P. and S.H. performed AFM nanoindentation measurements. All the authors discussed the results and commented on the manuscript. W.L., S.W., and H.C. supervised the research.

## Conflicts of Interest

The authors declare no conflict of interest.

## Supporting information




**Supporting File**: adma71968‐sup‐0001‐SuppMat.docx.

**Supporting File**: adma71968‐sup‐0002‐MovieS1.mp4.

## Data Availability

The data that support the findings of this study are available from the corresponding author upon reasonable request.
