# Supplementary material for: Hybrid Liquid Metal Cathode Enables High‐Performance Intrinsically Stretchable OLEDs
Source: Adv Mater. 2025 Dec 28;38(25):e18254. doi: 10.1002/adma.202518254 (PMC13137763; doi:10.1002/adma.202518254)
Supplement: Supplementary file 1 — Supporting File: adma71968‐sup‐0001‐SuppMat.docx. [file ADMA-38-e18254-s001.docx]

Supplementary Information for

**Hybrid Liquid Metal Cathode Enables High-Performance Intrinsically Stretchable OLEDs**

Wonbeom Lee^1,2^, Wei Liu^2,3,^*, Cheng Zhang^2^, Seungmin Shin^1^, Jaejun Lee^1^, Jaedong Jang^1^, Sanggil Park^1^, Seungbum Hong^1^, Sihong Wang^2,^*, Himchan Cho^1,4,^*

^1^ Department of Materials Science and Engineering, Korea Advanced Institute of Science and Technology (KAIST), Daejeon, Republic of Korea.

^2^ Pritzker School of Molecular Engineering, The University of Chicago, Chicago, IL, USA.

^3^ Institute of Functional Nano & Soft Materials (FUNSOM), Soochow University, Suzhou, Jiangsu, China.

^4^ Graduate School of Semiconductor Technology, School of Electrical Engineering, Korea Advanced Institute of Science and Technology (KAIST), Daejeon, Republic of Korea.

* To whom correspondence should be addressed:

E-mail: [wei6@suda.edu.cn](mailto:wei6@suda.edu.cn) (W. L.); [sihongwang@uchicago.edu](mailto:sihongwang@uchicago.edu) (S. W.); [himchan@kaist.ac.kr](mailto:himchan@kaist.ac.kr) (H. C.)


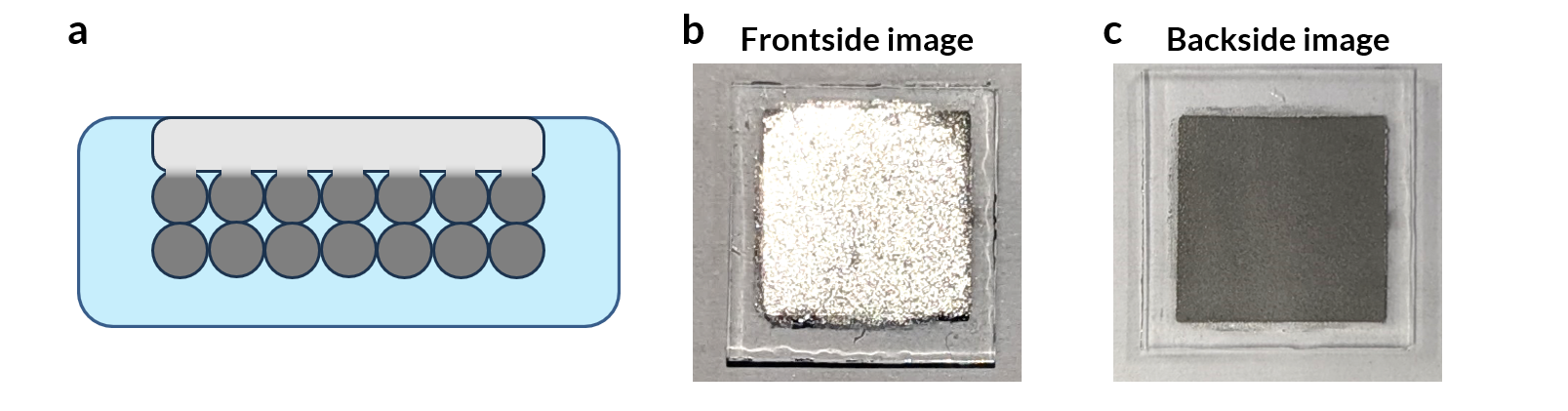
 **Supplementary Fig.** **1** | **Morphology of Hybrid liquid metal (Hyb-LM).**

**a**, Schematic illustration of Hyb-LM. **b**, **c**, Photographs of (**b**) front side and (**c**) backside of Hyb-LM. The front side of Hyb-LM is glossy as the surface is comprised of LM and backside of Hyb-LM is gray-colored because of the underlying liquid metal particles (LMPs).


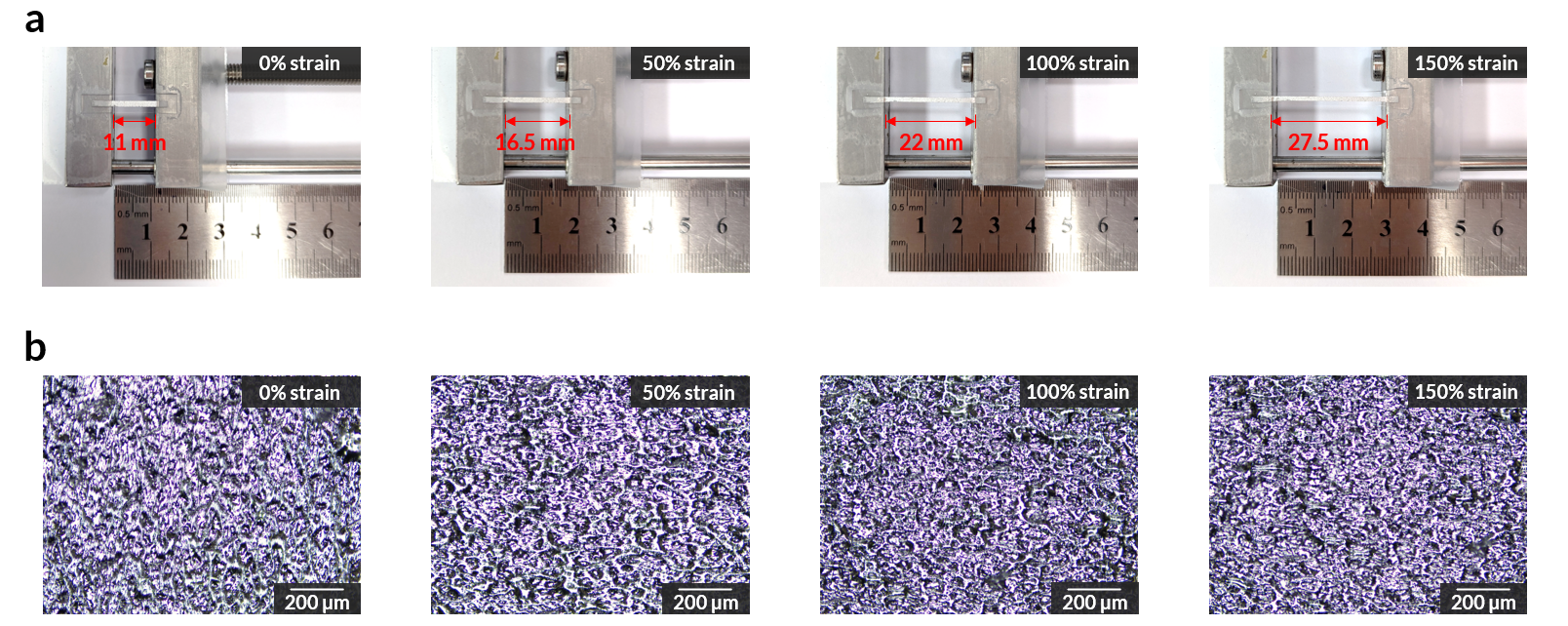
**Supplementary Fig. 2 | Photographs and optical microscope images of Hyb-LM under deformation.**

**a**, Photographs of Hyb-LM under 0%, 50%, 100% and 150% strain, showing high stretchability of Hyb-LM up to 150%. **b**, Optical microscope image of Hyb-LM under 0%, 50%, 100% and 150% strain, showing the liquid nature of surface of Hyb-LM under strain.


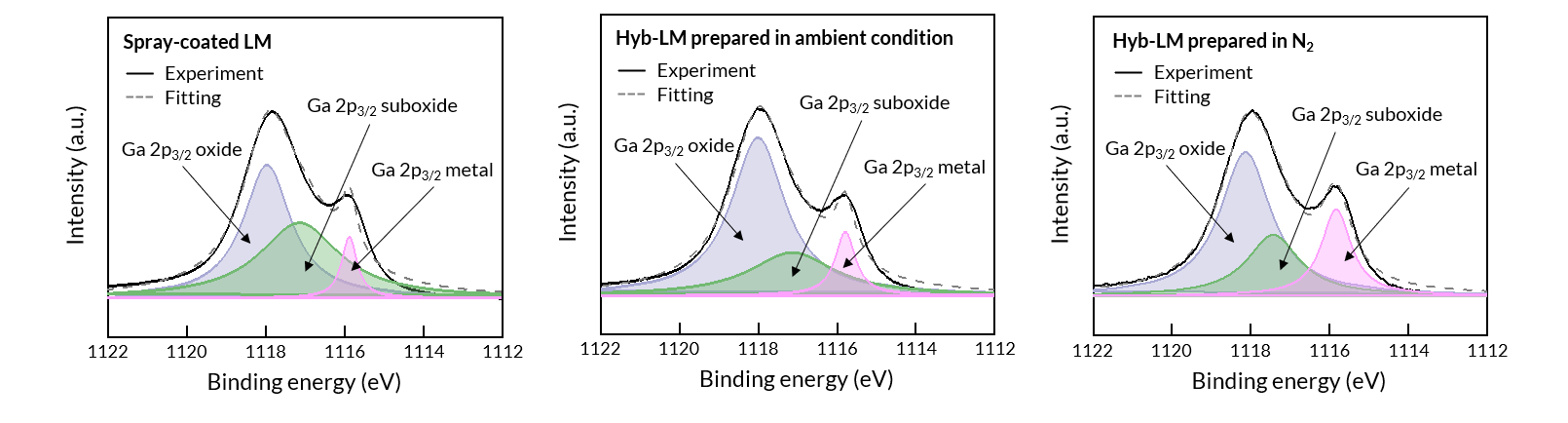


**Supplementary Fig. 3 | XPS measurements of spray-coated LM, and Hyb-LM electrodes.**

Hyb-LM prepared in N_2_ atmosphere shows substantial increase in metallic Ga proportion (pink) compared to spray-coated LM and Hyb-LM prepared in ambient conditions.


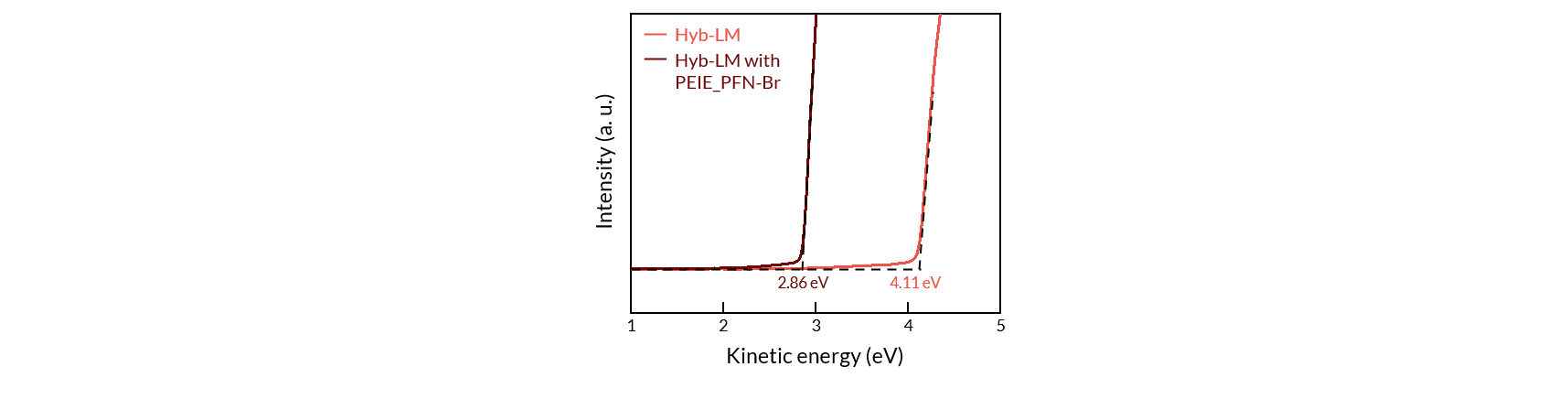


Supplementary Fig. 4 | Work function of Hyb-LM and Hyb-LM with PEIE_PFN-Br.

UPS spectra of Hyb-LM (4.11 eV, bright red) and Hyb-LM with PEIE_PFN-Br (2.86 eV, dark red), showing a low work-function of Hyb-LM electrode.


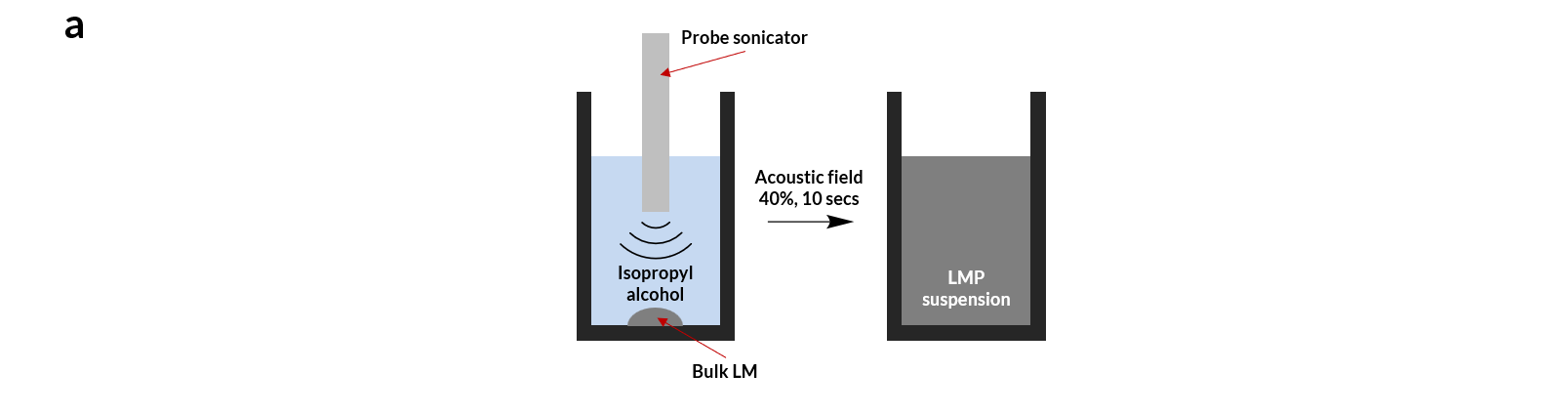

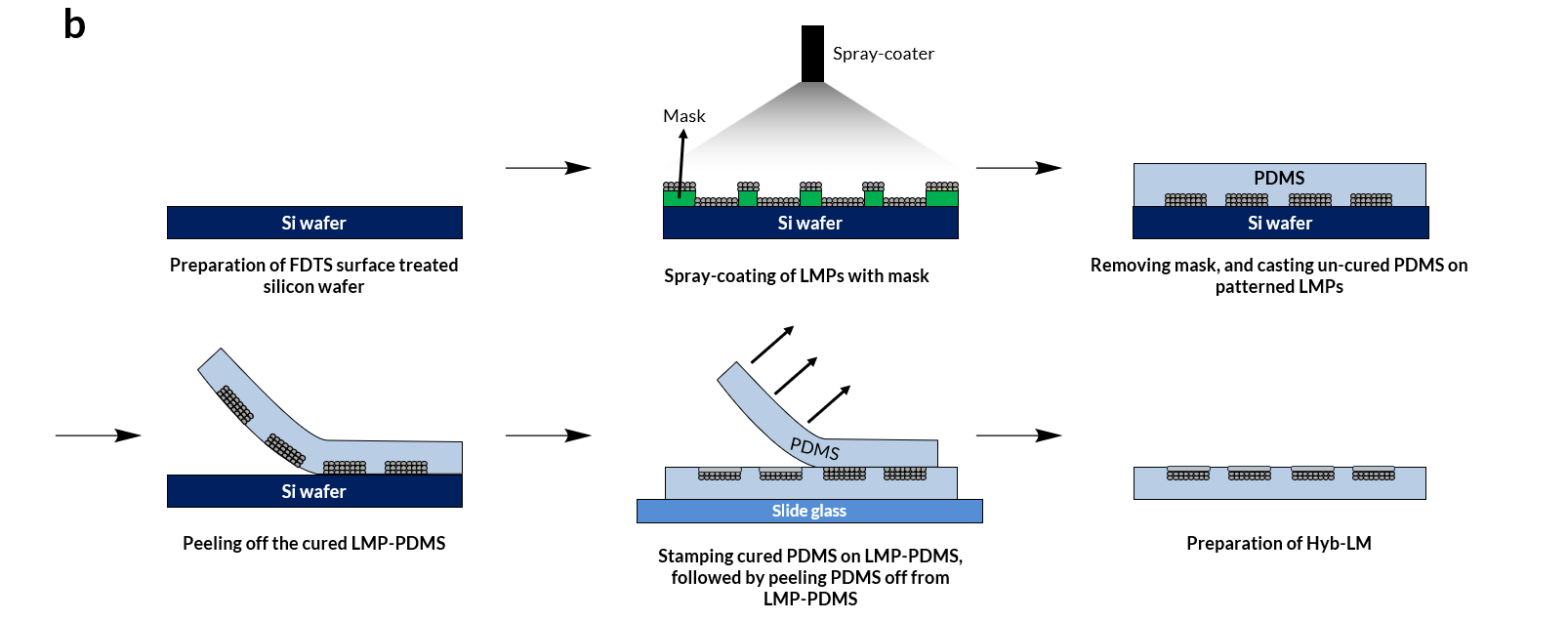
Supplementary Fig. 5 | Detailed procedure of preparation of Hyb-LM.

**a**, Schematic illustration of preparation of LMP solution for spray-coating process. **b**, Schematic illustration of preparation of Hyb-LM electrode.


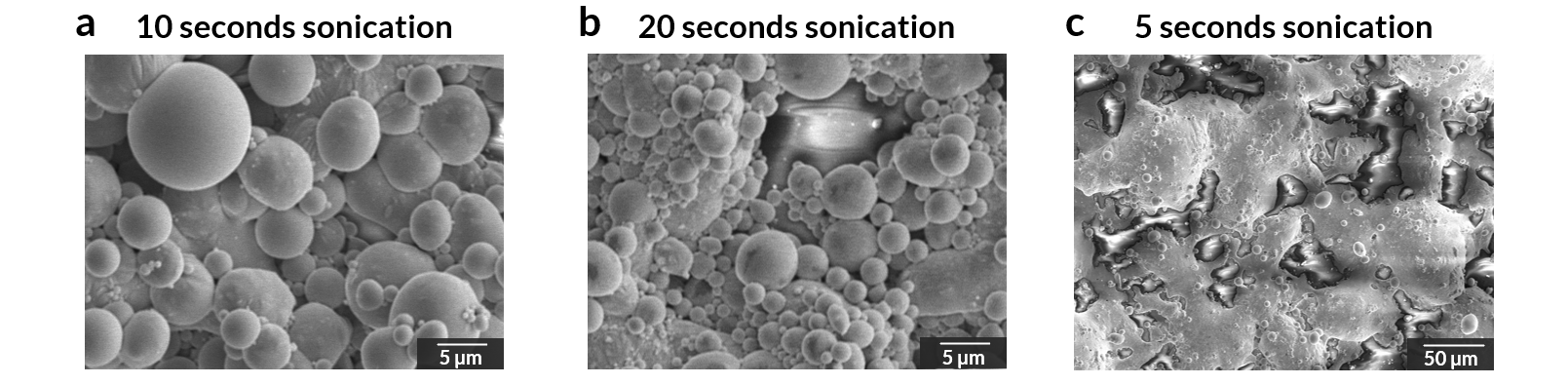
 Supplementary Fig. 6 | SEM images of spray-coated LMPs on glass substrates.

LMP particles prepared with 5 seconds of probe sonication coalesced into bulk LM during spray-coating, likely due to the high air pressure involved in the process. In contrast, particles prepared with 10 and 20 seconds of probe sonication retained their discrete morphologies after deposition. The particle size distribution varied with sonication time: those obtained from 10 seconds of sonication were predominantly larger than 4 µm, whereas those from 20 seconds of sonication were mainly smaller than 2 µm, with a significant fraction reaching the nanoscale.


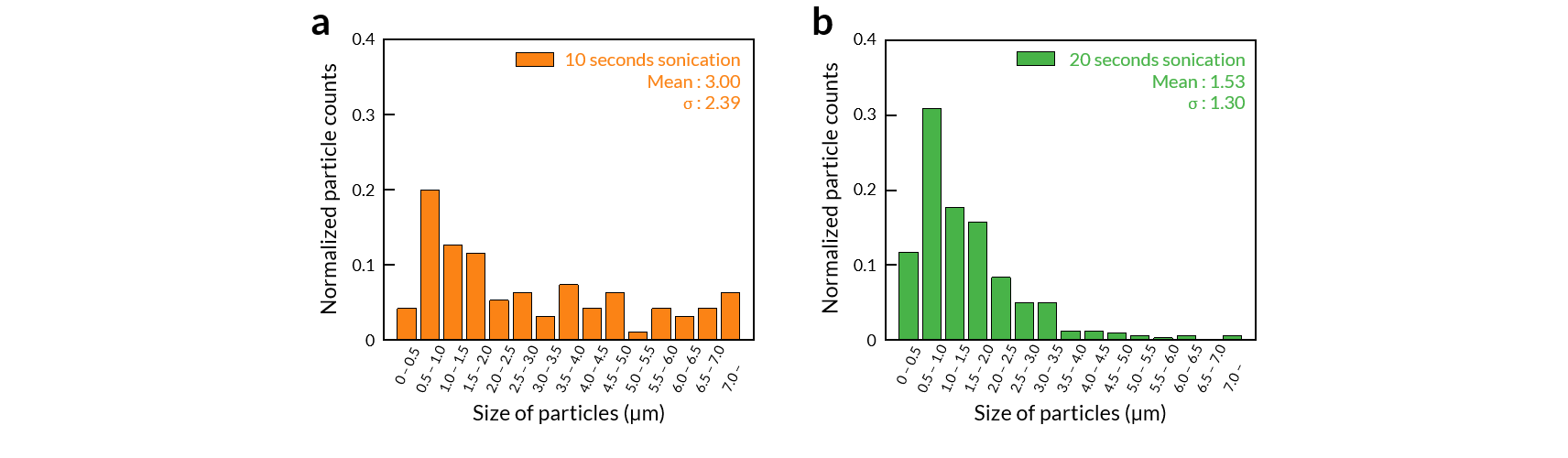


Supplementary Fig. 7 | Particle size distribution of spray-coated LMPs.

**a**, **b**, Particle size distribution of LMPs with (**a**) 10 seconds of sonication and (**b**) 20 seconds of sonication. Size of particles were measured using Image-J software.


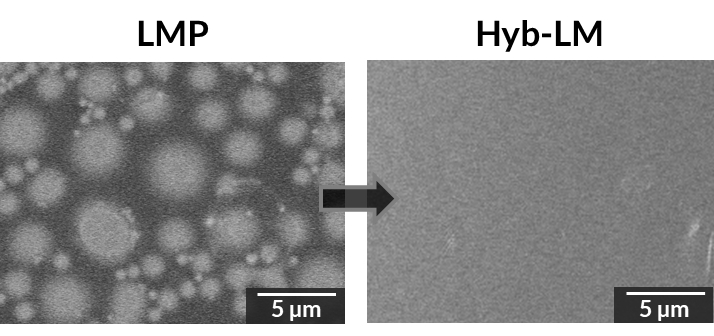


Supplementary Fig. 8 | SEM images of LMP and Hyb-LM.

SEM images of LMP and Hyb-LM, demonstrating the transformation of surface LMPs into a continuous LM layer through selective rupture.


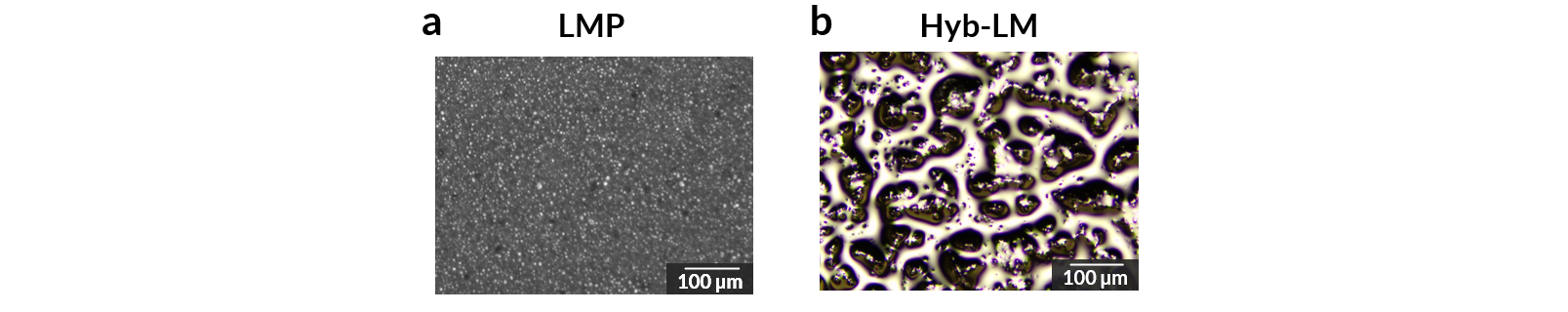


Supplementary Fig. 9 | Optical Microscope image of liquid metal particles (LMP) and hybrid liquid metal (Hyb-LM).

**a**, **b**, Optical microscope images of (**a**) LMP, (**b**) Hyb-LM, showing that the surface of Hyb-LM comprises of bulk LM, in contrast to LMP.


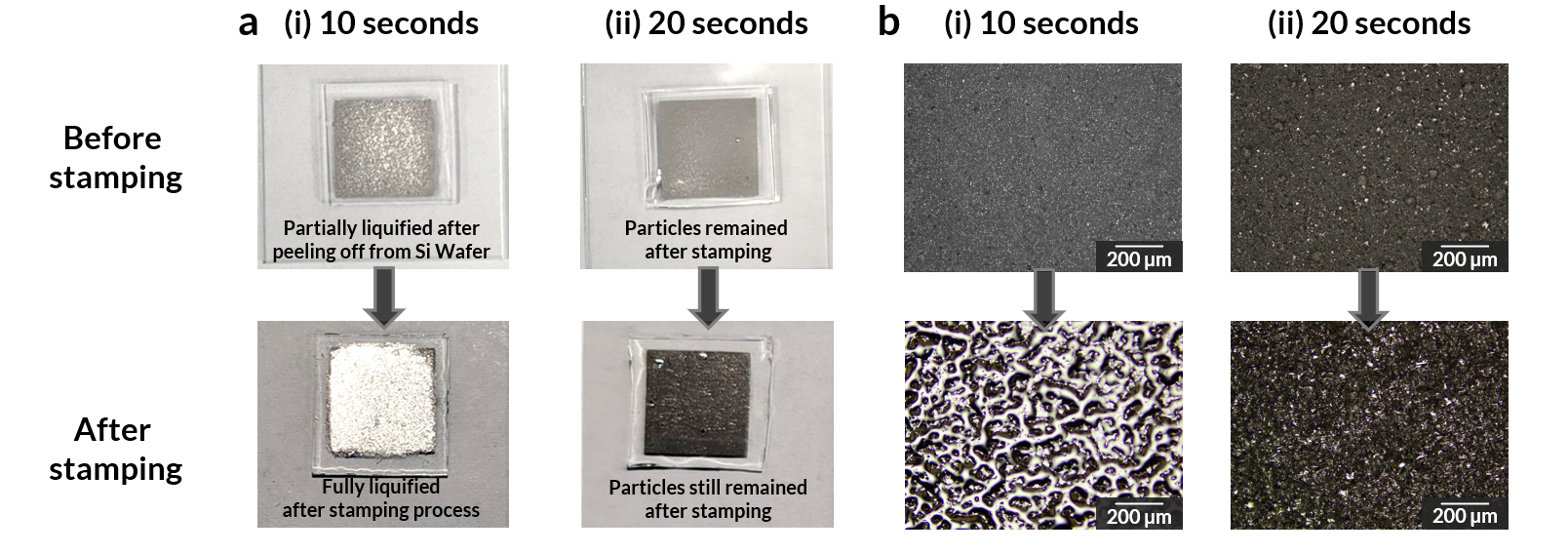
 Supplementary Fig. 10 | Effects of sonication time on the preparation of Hyb-LM.

**a**, **b**, (**a**) Photographs and (**b**) Optical microscope images of the electrodes with different sonication time, before and after stamping process.


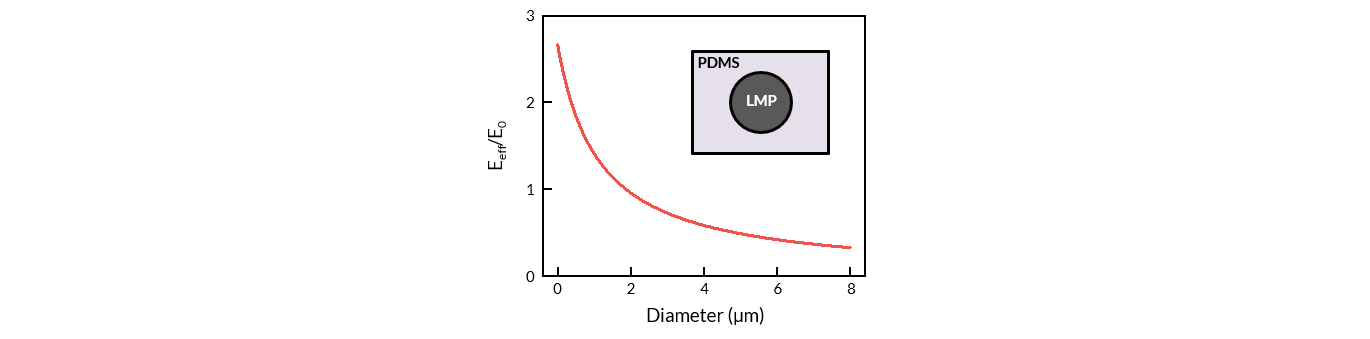


Supplementary Fig. 11 | Predicted effective Young’s modulus of the LMPs.

Predicted effective Young’s modulus of LMPs from generalized Eshelby theory, E_eff_ = E_0_$\frac{24\gamma/E_{0}R}{10+9\gamma/E_{0}R}$. Young’s modulus (E_0_), surface tension are set to 1 MPa, 624 mN/m, respectively. The graph shows that effective young’s modulus decreases as the size of particle increases.


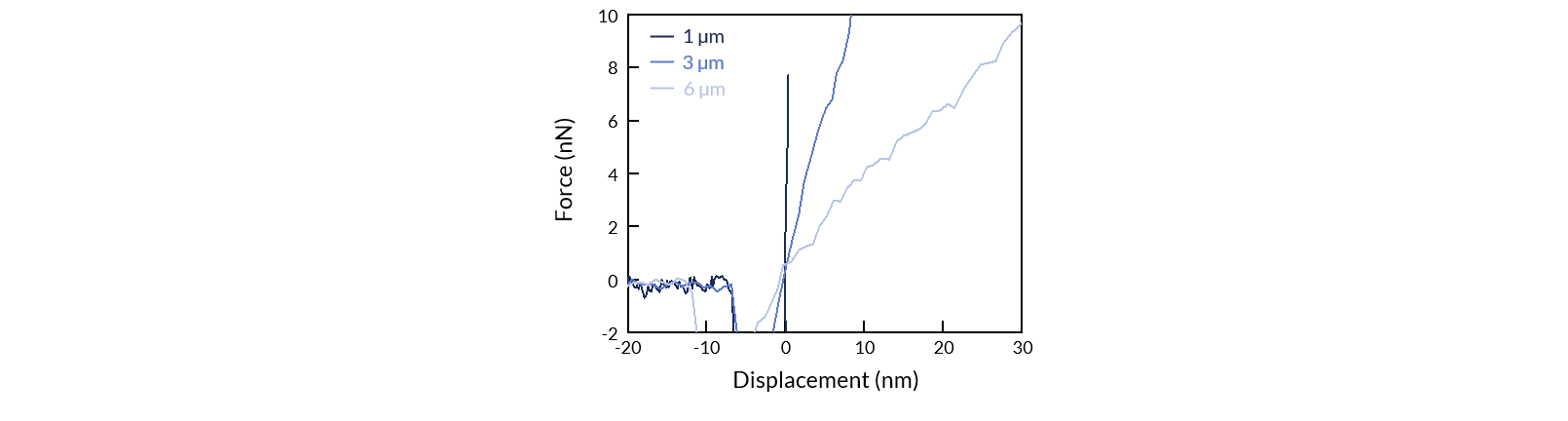


**Supplementary Fig. 12 | Mechanical response of LMPs with different sizes.**

Nanoindentation measurements using AFM on LMPs with diameters of 1 µm, 3 µm, and 6 µm. The elastic moduli of LMPs were calculated to be 1.92 GPa, 21.35 MPa, 3.84 MPa, respectively, showing a substantial decrease in stiffness with increasing particle size.


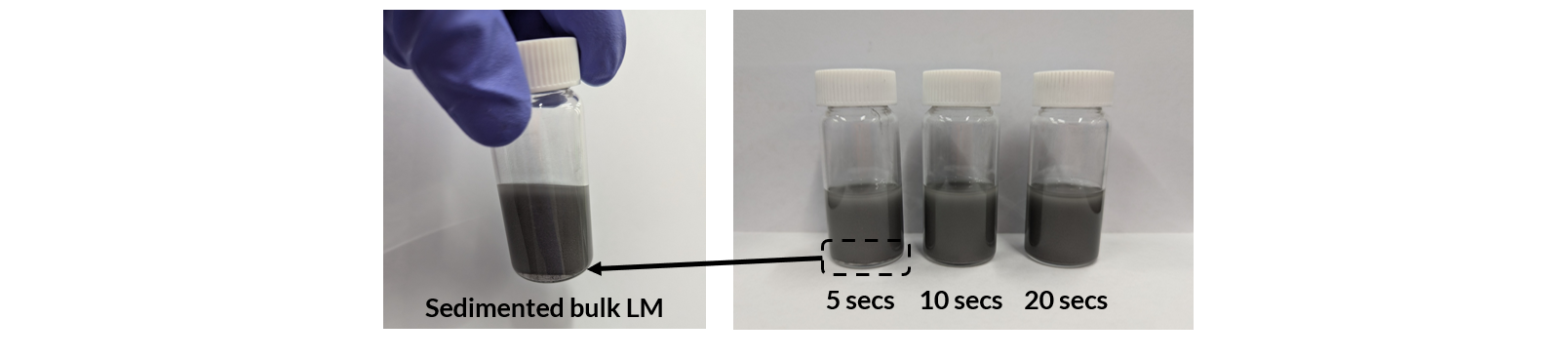
 Supplementary Fig. 13 | Stability of LMP dispersions under shaking.

LMPs in the dispersions sonicated for 10 and 20 seconds remained stable upon manual shaking. In contrast, LMPs in the dispersion sonicated for 5 seconds coalesced into bulk LM after shaking, indicating insufficient dispersion stability with agitation.


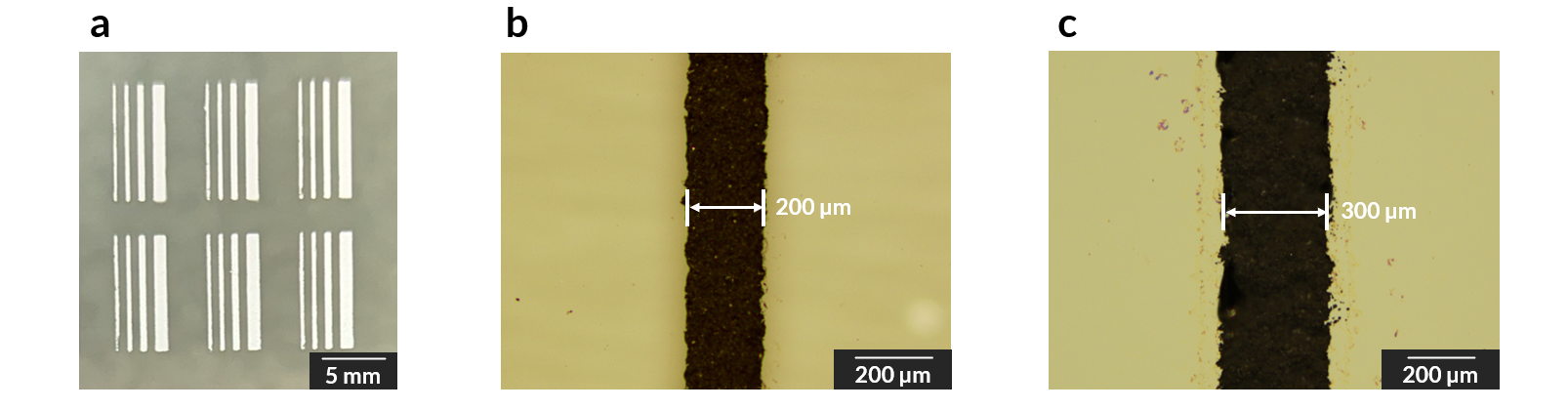
 Supplementary Fig. 14 | Patterned LMPs spray-coated on the wafer.

**a**, Photographs of spray-coated LMPs on the Si wafer that had been treated with FDTS. **b**, **c**, Optical microscope images of LMP lines with (**b**) 200 µm width and (**c**) 300 µm width.


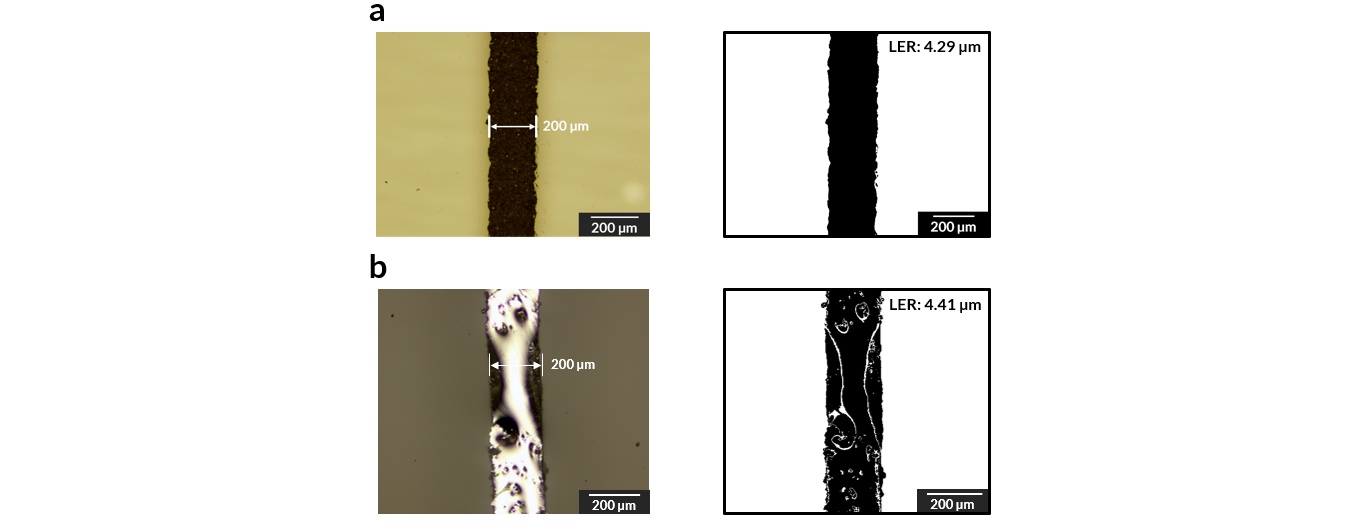


Supplementary Fig. 15 | Line edge roughness of patterned LMP and Hyb-LM.

**a**, **b**, Line edge roughness of (**a**) LMP and (**b**) Hyb-LM, showing the feasible patternability of Hyb-LM. The comparable line edge roughness of LMP (4.29 µm) and Hyb-LM (4.41 µm) confirm that the stamping process doesn’t compromise pattern fidelity. Line edge roughness was quantified using Fiji, Image J-based software. Optical microscope images were first converted to monochrome images, followed by extracting the x-axis position of the pattern edge along each y-axis line. The standard deviation of these edge positions was then calculated and converted from pixels to physical length, which is defined as the line edge roughness.


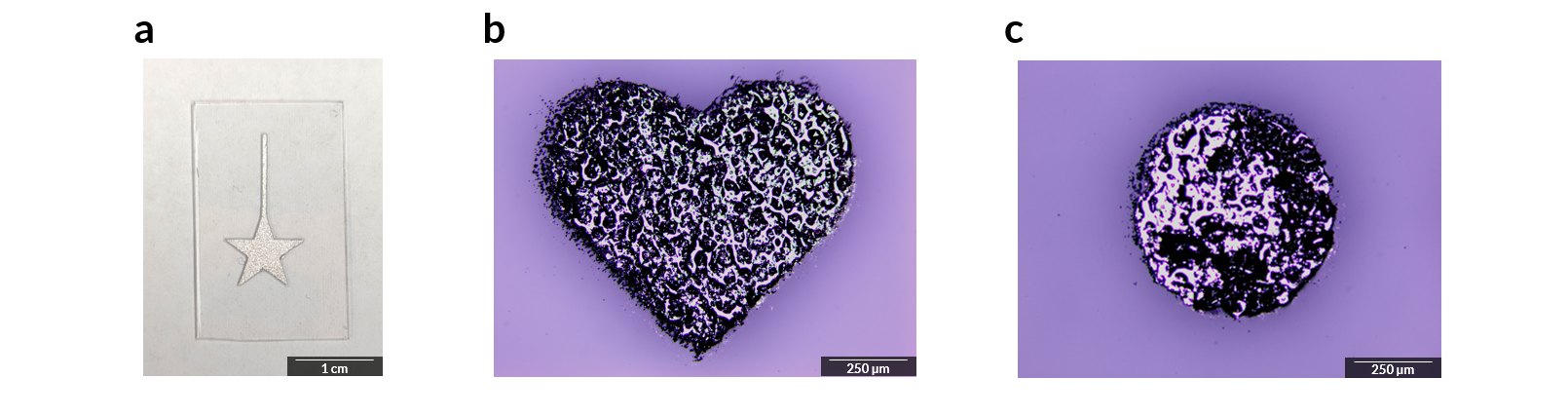


Supplementary Fig. 16 | Hyb-LM with various patterns.

a, Photograph of Hyb-LM in star shape. b, c, Optical microscope image of Hyb-LM in (b) heart and (c) dot shape.


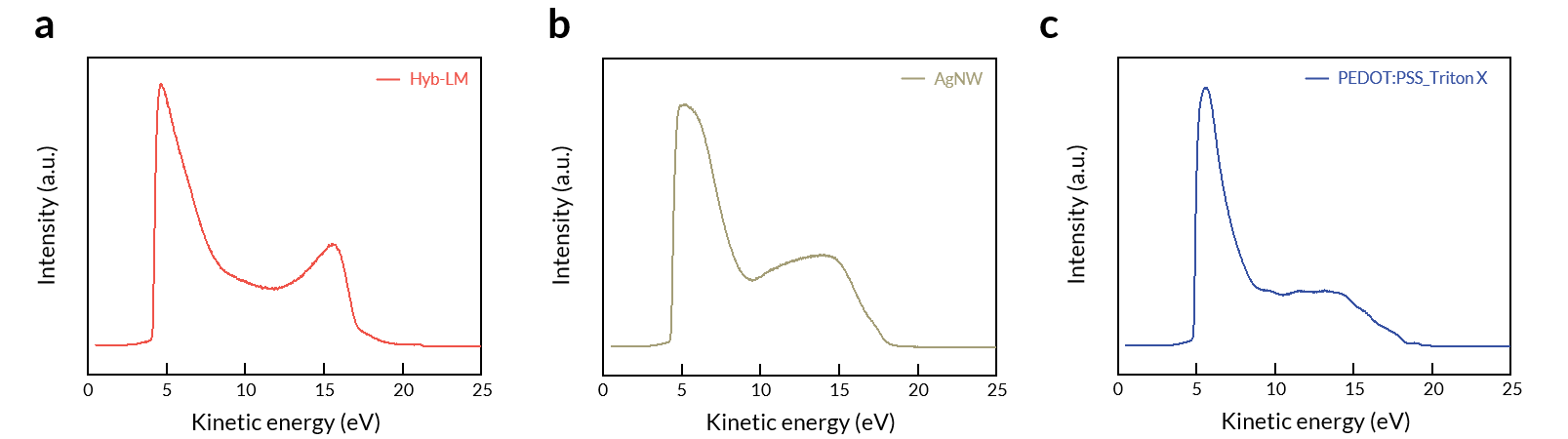
 Supplementary Fig. 17 | UPS measurement of stretchable electrodes.

**a**, **b**, **c**, UPS spectra of stretchable electrodes: (**a**) Hyb-LM, (**b**) AgNW and (**c**) PEDOT:PSS_Triton X. The results show that Hyb-LM exhibits the lowest work function, suggesting its suitability as a stretchable cathode.


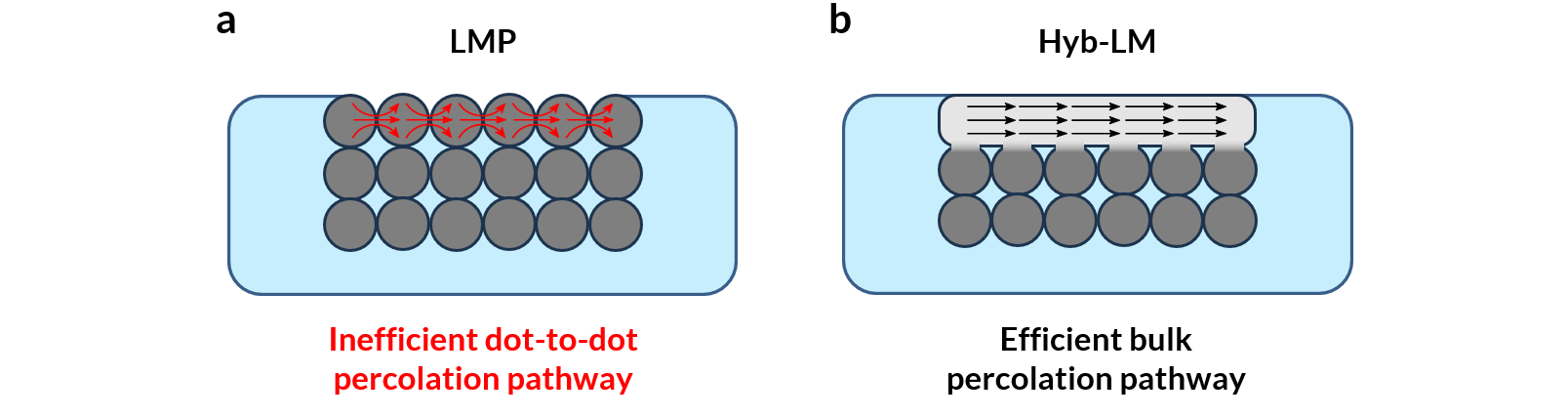
 Supplementary Fig. 18 | Schematic illustrations of percolation pathways in LMP and Hyb-LM.

**a**, LMP exhibits limited percolation pathways due to point contacts between discrete particles.
**b**, In contrast, Hyb-LM forms continuous percolation pathways across the surface of the bulk LM, resulting in a lower sheet resistance compared to LMP.


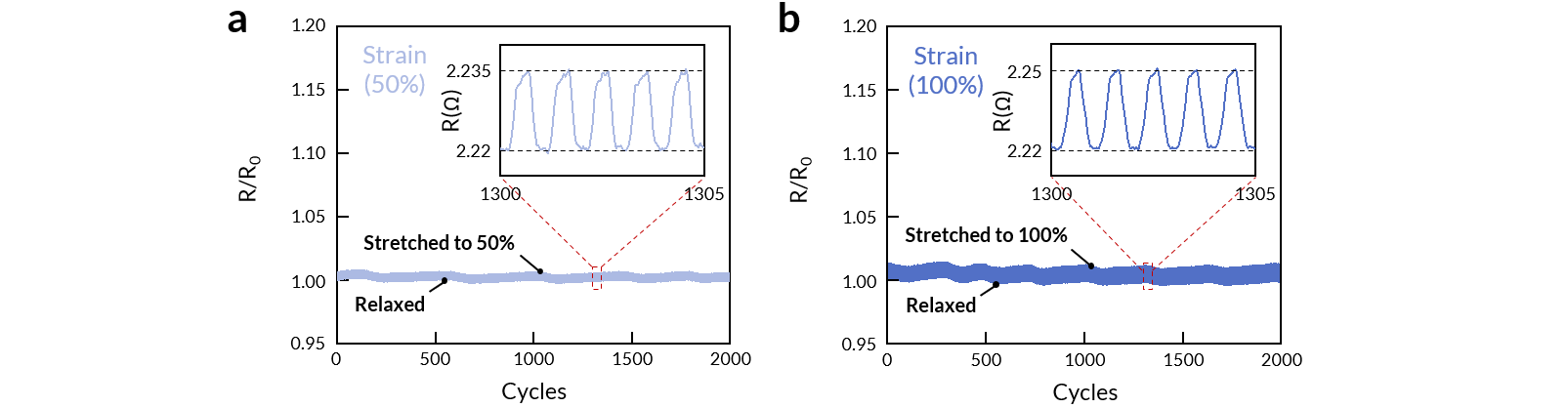


Supplementary Fig. 19 | Cyclic stability of Hyb-LM under different strains.

**a**, **b**, Relative change in resistance of Hyb-LM under (**a**) 50% and (**b**) 100% cyclic strain up to 2000 cycles. The insets show detailed resistance responses to the applied strain during cycling.


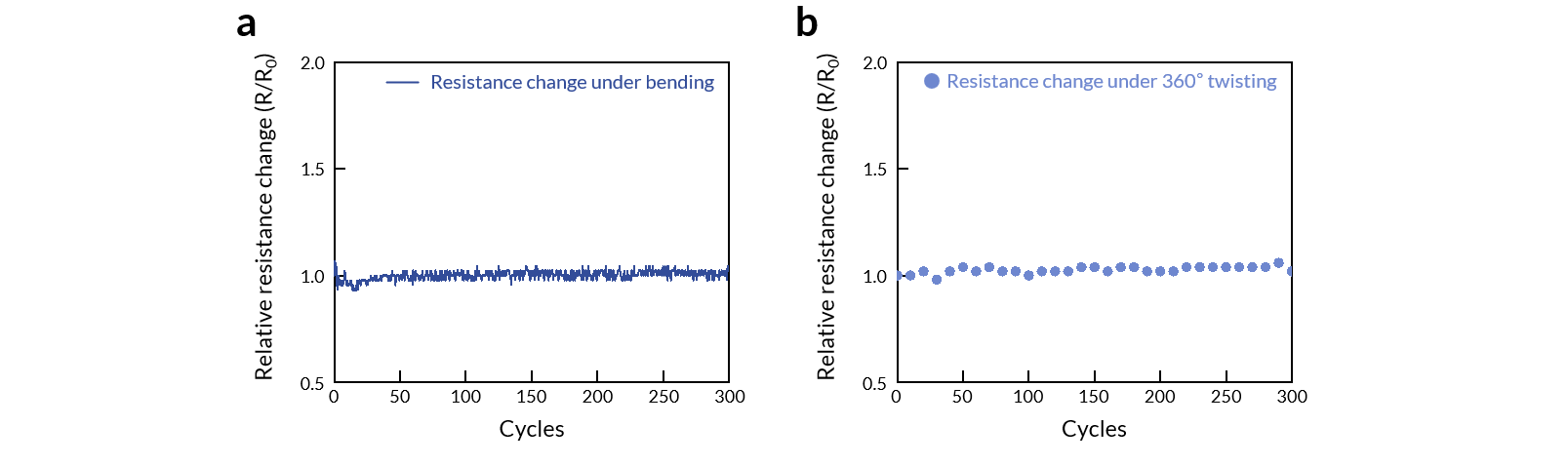


**Supplementary Fig. 20 | Cyclic stability of Hyb-LM under bending and 360° twisting.**

Relative change in resistance of Hyb-LM under (**a**) bending and (**b**) 360° twisting up to 300 cycles, respectively.


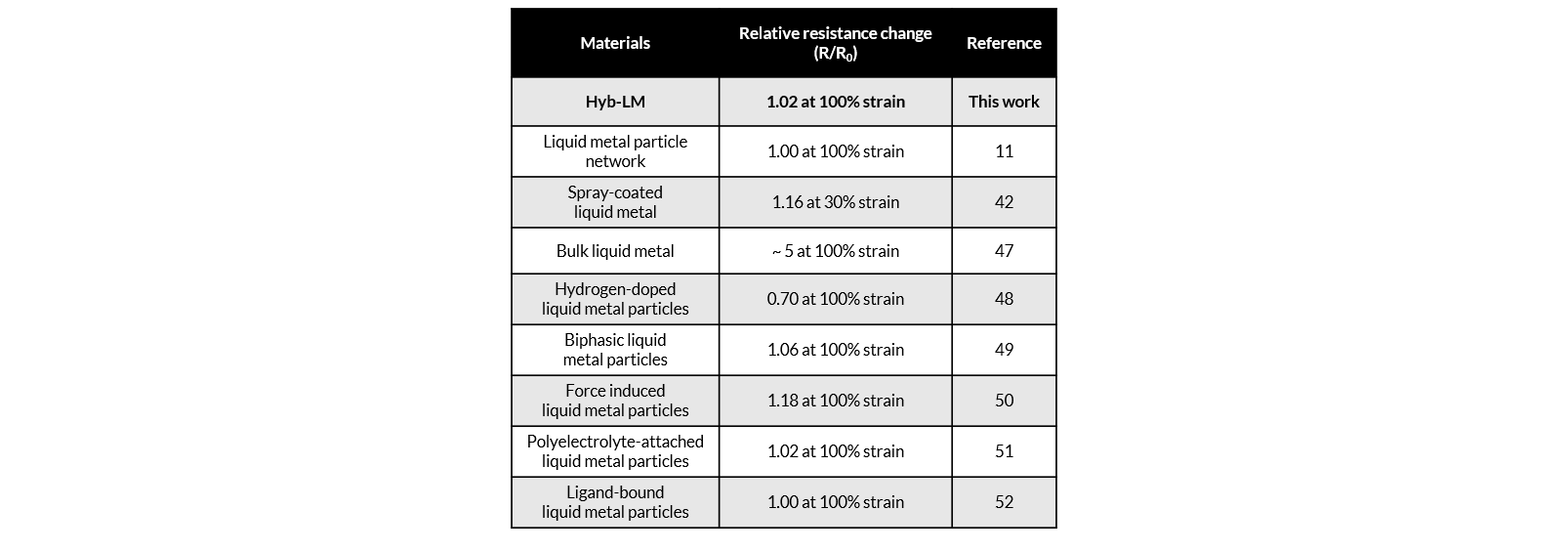


**Supplementary Table 1 | Comparison of the electromechanical properties** **of Hyb-LM with the previously reported liquid metal-based stretchable conductors.**


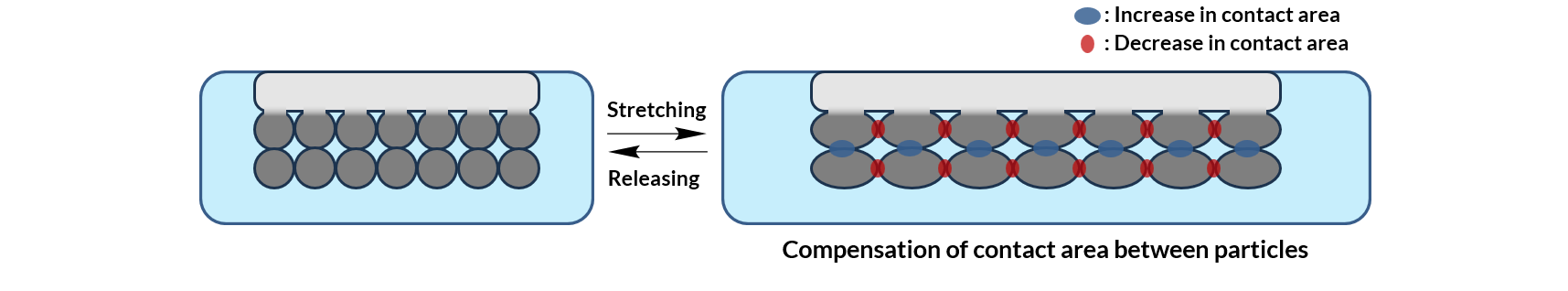


Supplementary Fig. 21 | Schematic illustration of Hyb-LM under strain.

Schematic illustration of Hyb-LM under strain, showing that resistance of Hyb-LM remains constant due to the compensation of contact area of underlying liquid metal particles under strain.


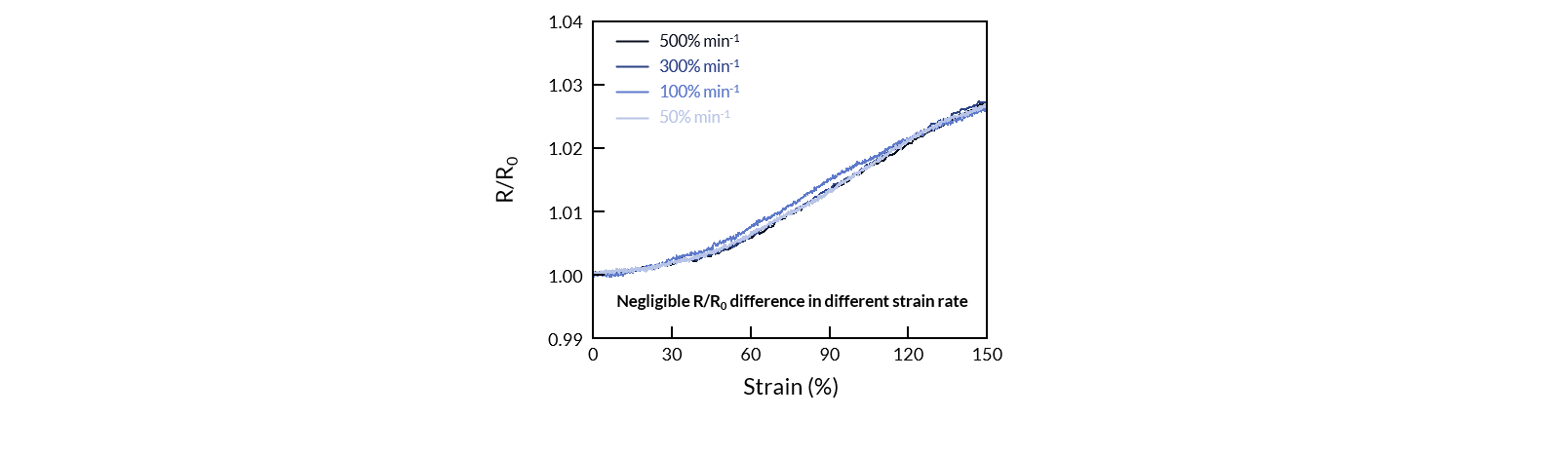


Supplementary Fig. 22 | Relative resistance changes of Hyb-LM under different strain rates.

Relative resistance changes of Hyb-LM under 150% uniaxial stretching at different strain rates (50% min^-1^, 100% min^-1^, 300% min^-1^, and 500% min^-1^), demonstrating that resistance change of Hyb-LM is irrelevant to the applied strain rate.


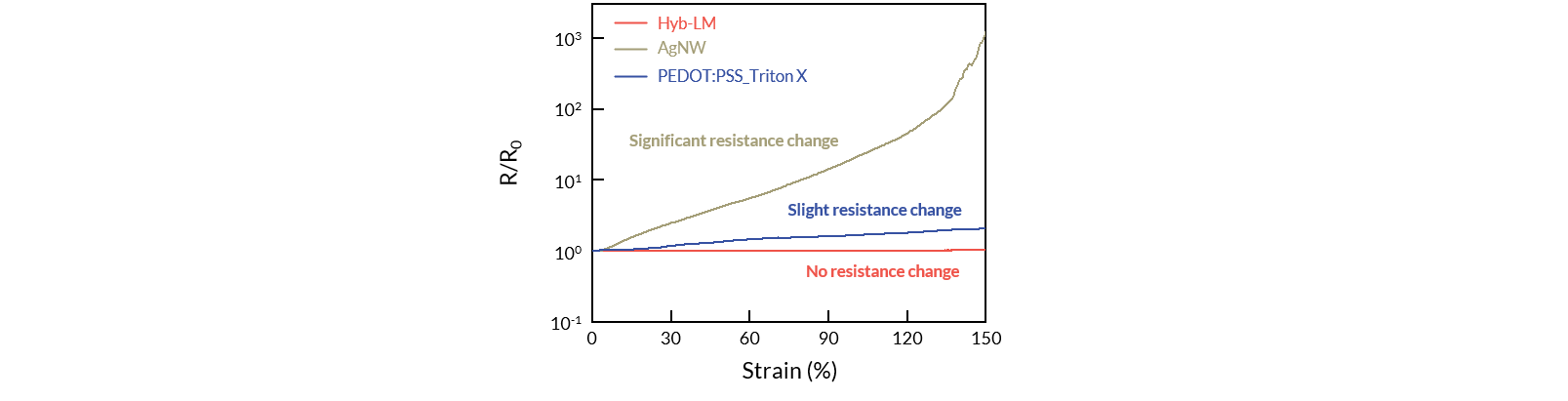


Supplementary Fig. 23 | Superior electromechanical performance of Hyb-LM.

Relative resistance changes of Hyb-LM, PEDOT:PSS_Triton X, and AgNW under 150% uniaxial strain. Hyb-LM showed negligible resistance change (R/R_0_ = 1.03 at 150% strain), whereas PEDOT:PSS_Triton X showed a moderate increase (R/R_0_ = 2.06 at 150% strain). In contrast, AgNW showed a drastic increase in resistance (R/R_0_ = 1211 at 150% strain). These results demonstrate the superior electromechanical performance of Hyb-LM compared to other stretchable electrodes.


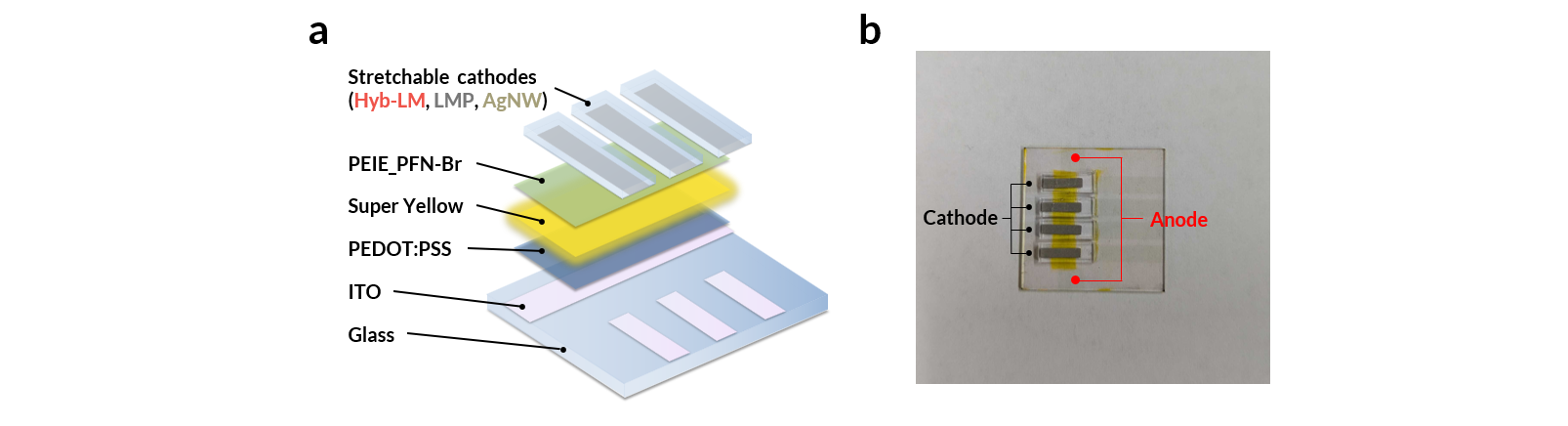


Supplementary Fig. 24 | Structure of rigid SY OLED.

**a**, Schematic illustration of rigid OLED with stretchable cathodes. **b**, Photographs of rigid OLED using SY as an emission layer. Black and red dots indicate the cathode and anode positions on the patterned ITO substrate, respectively. Note that the separated ITO patterns on the right-hand side of the glass substrate are used as contact pads for stretchable cathodes.


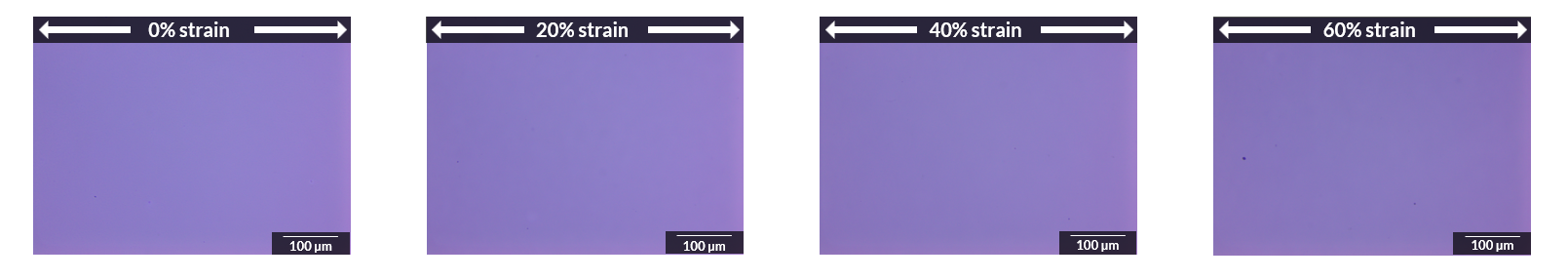


**Supplementary Fig.** **25 | Stretchability of PEIE_PFN-Br.**

Optical microscope images of PEIE_PFN-Br film with varying uniaxial strain from 0% to 60%. No cracks were observed up to 60% strain. The stretching direction is horizontal.


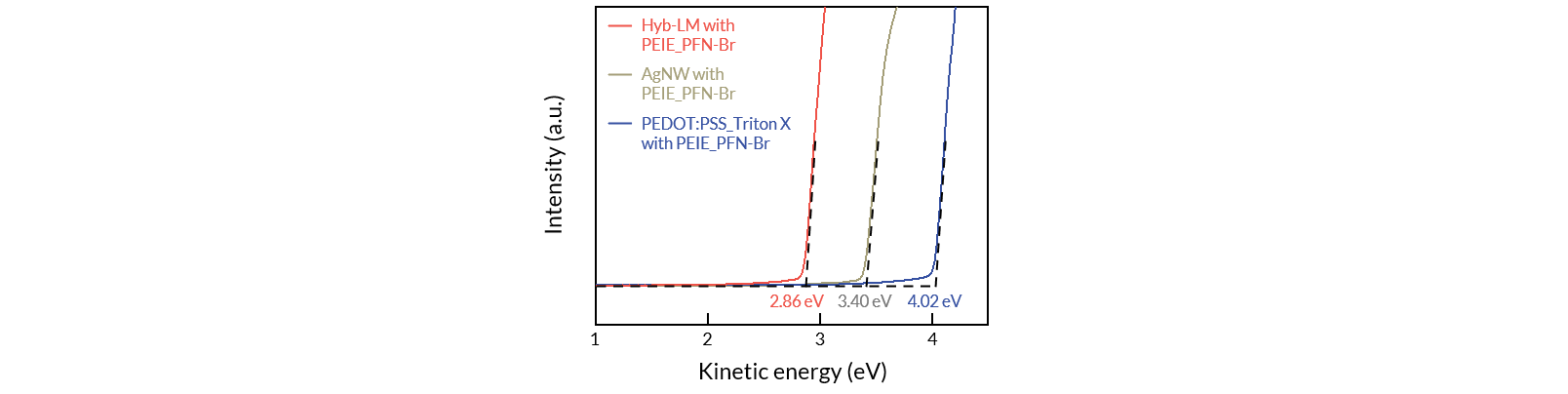


Supplementary Fig. 26 | UPS spectra of PEIE_PFN-Br coated stretchable electrodes.

The UPS spectra show the work functions of electrcodes after spin-coating of PEIE_PFN-Br: Hyb-LM with PEIE_PFN-Br (2.86 eV, red), AgNW with PEIE_PFN-Br (3.40 eV, olive green), and PEDOT:PSS_Triton X with PEIE_PFN-Br (4.02 eV, blue). The significantly lower work function of PEIE_PFN-Br coated Hyb-LM indicates its superior capability for efficient electron injection.


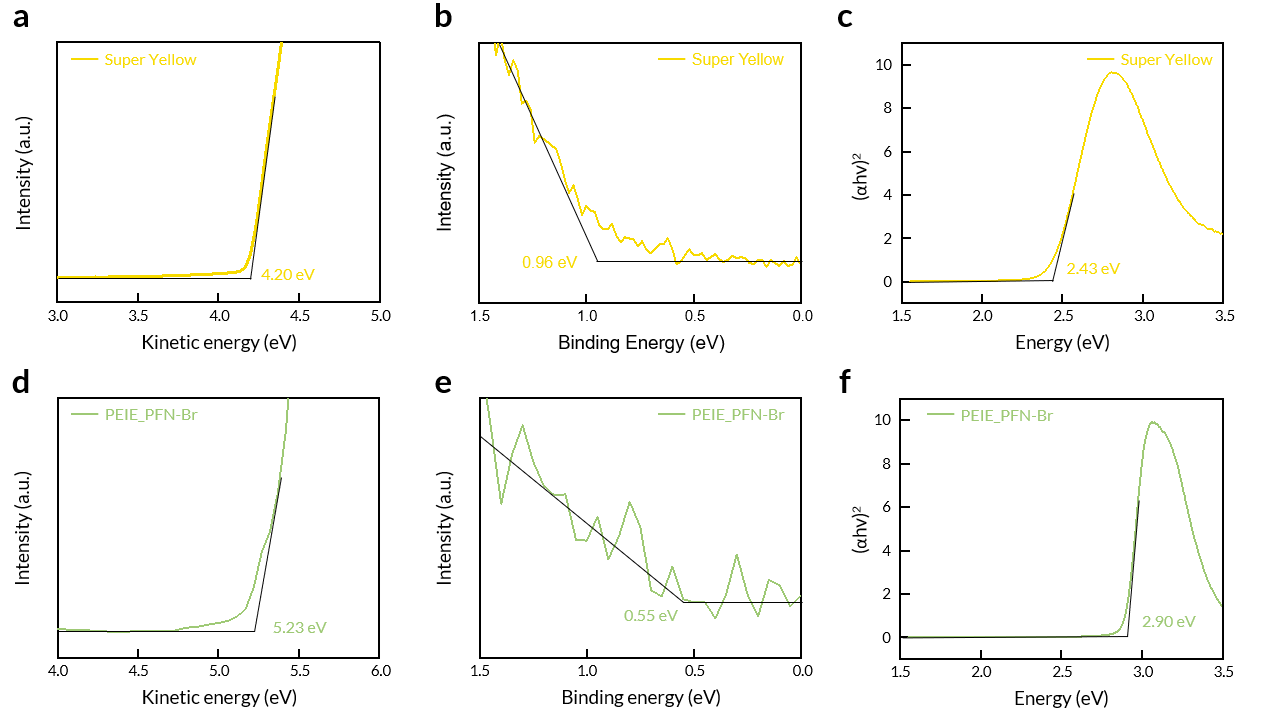

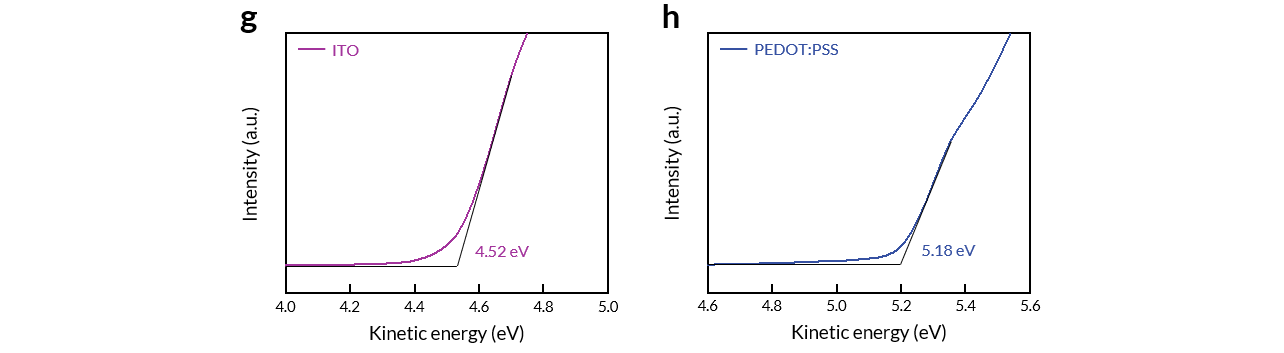
 Supplementary Fig. 27 | Energy level characterization of materials used in the SY-based rigid OLED.

**a**, **b**, UPS spectra of SY film coated on ITO substrate, showing (**a**) secondary cut-off and (**b**) highest occupied molecular orbital (HOMO) edge. **c**, Tauc plot of SY film. **d**, **e**, UPS spectra of PEIE_PFN-Br film coated on ITO substrate, showing (**d**) secondary cut-off and (**e**) HOMO edge. **f**, Tauc plot of PEIE_PFN-Br film. **g**, **h**, UPS spectra (secondary cut-off) of (**g**) ITO and (**h**) PEDOT:PSS (AI4083) films.


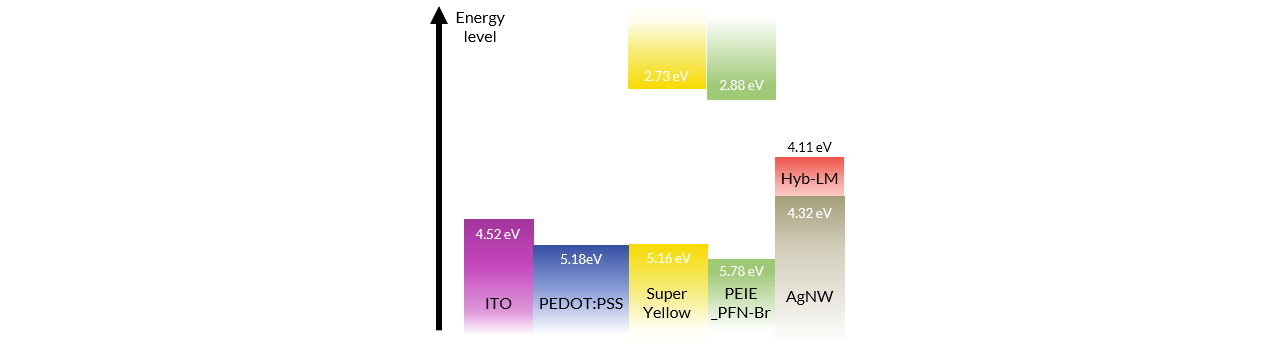


Supplementary Fig. 28 | Energy-level diagram of SY-based rigid OLED.


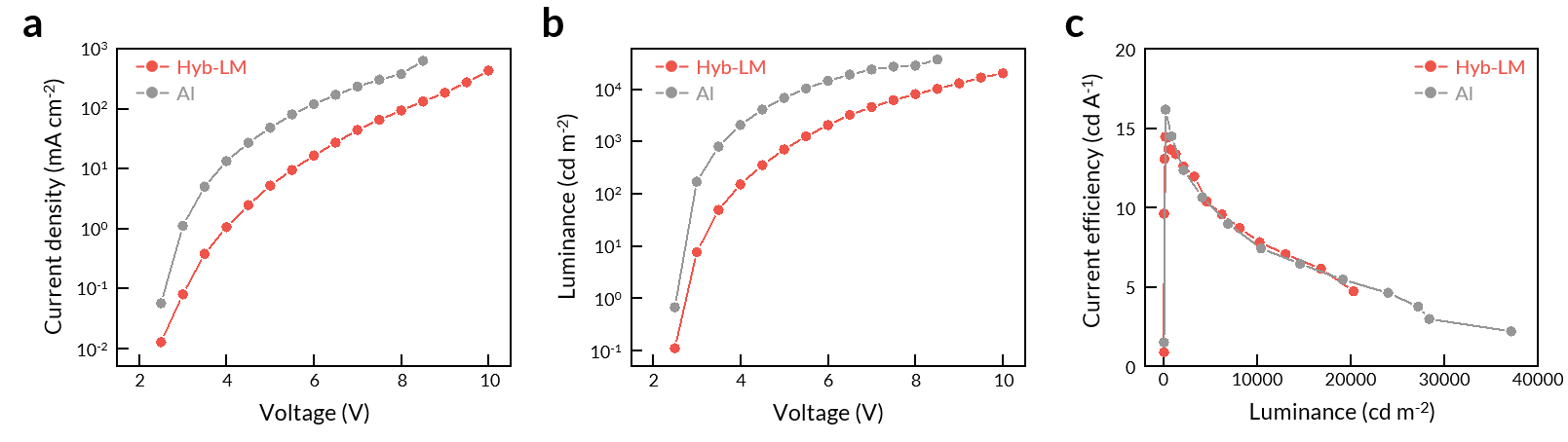
 **Supplementary Fig.** **29 | Comparison of Hyb-LM cathode to Al cathode with SY rigid OLED.**

**a**, **b**, **c**, (**a**) Current density, (**b**) luminance and (**c**) current efficiency of SY rigid OLED using Hyb-LM and thermally evaporated Al. The devices showed similar trends in current efficiency-luminance traces.


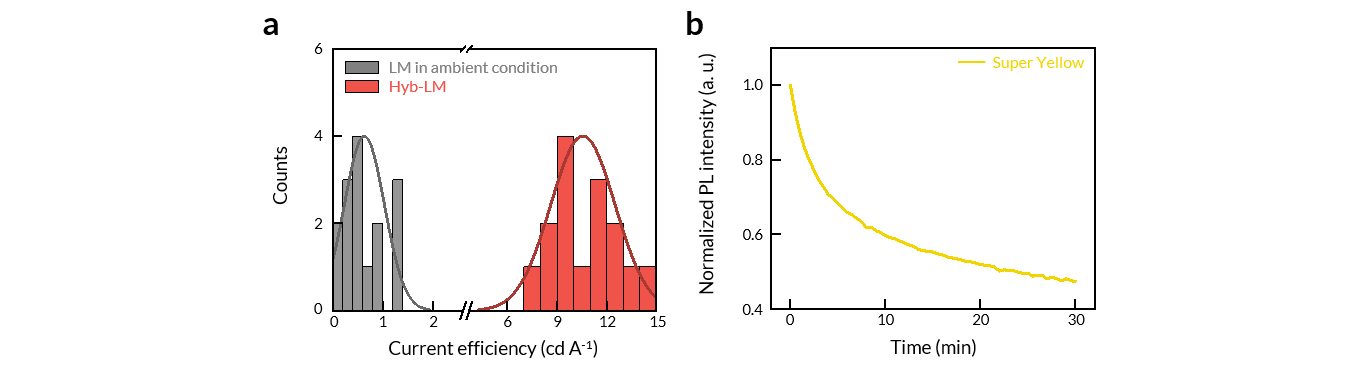


**Supplementary Fig.** **30 | Comparison of OLED with Hyb-LM cathode processed in oxygen-free condition to OLED with LM processed in ambient condition.**

**a**, Current efficiency histogram of OLED with Hyb-LM cathode processed in oxygen-free condition and OLED with LM processed in ambient condition (15 devices each). **b**, Normalized photoluminescence intensity of SY film in ambient condition. Normalized intensity decreases due to the non-radiative decay from the formation of trap levels in ambient condition.


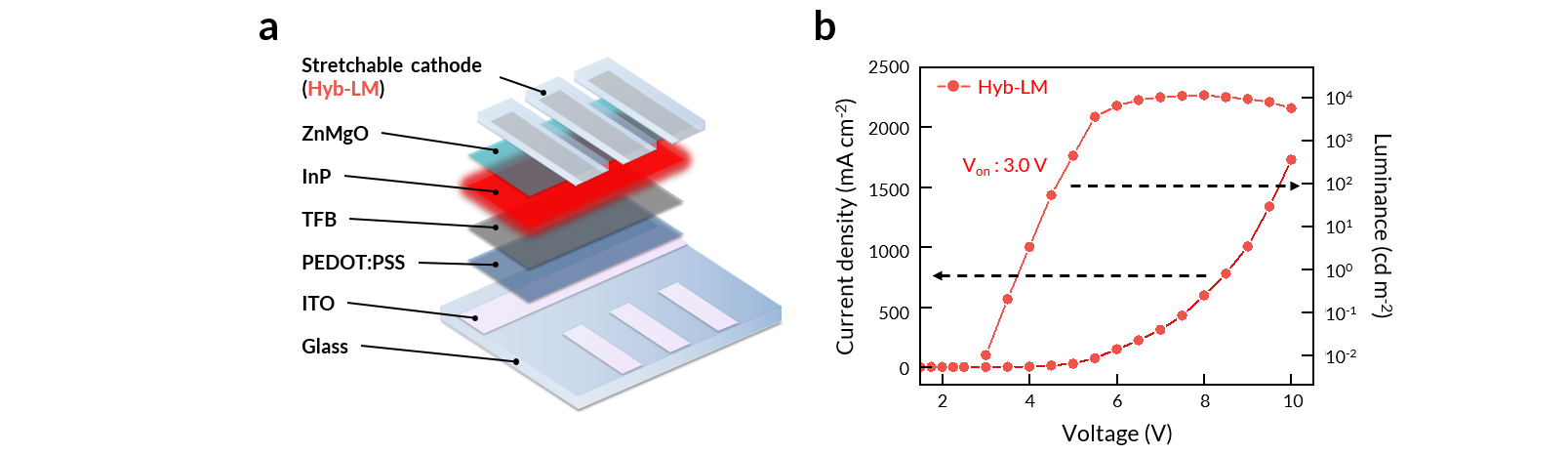


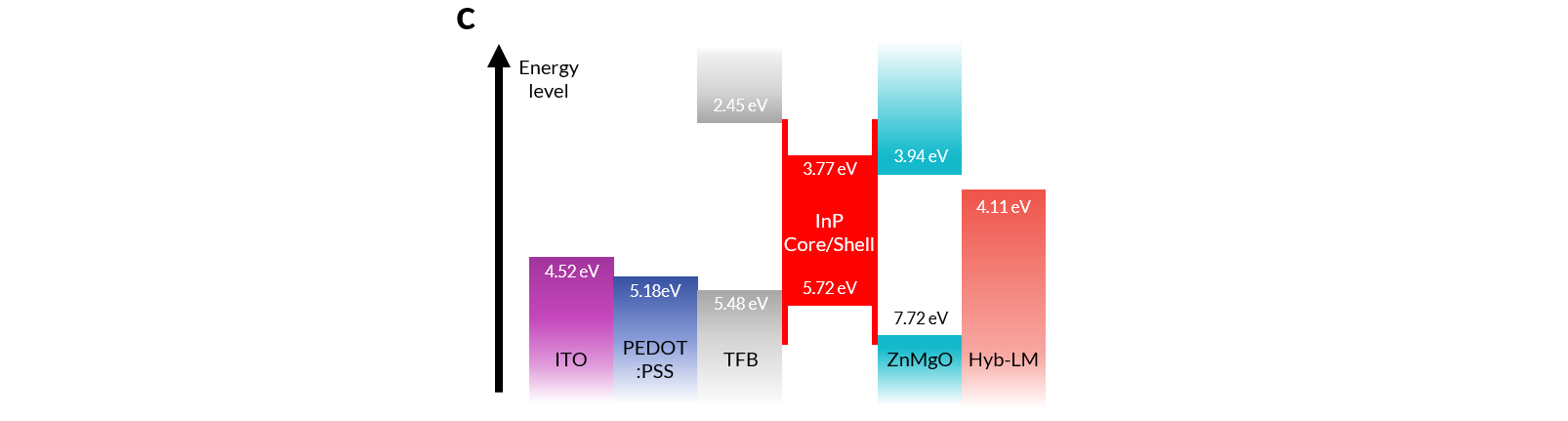
Supplementary Fig. 31 | InP QD-based rigid QLED.

**a**, Schematic illustration of rigid QLED using red InP-based core/shell QDs as an emission layer. **b**, Representative current density-luminance-voltage traces. **c**, Energy-level diagram of rigid QLED.

**
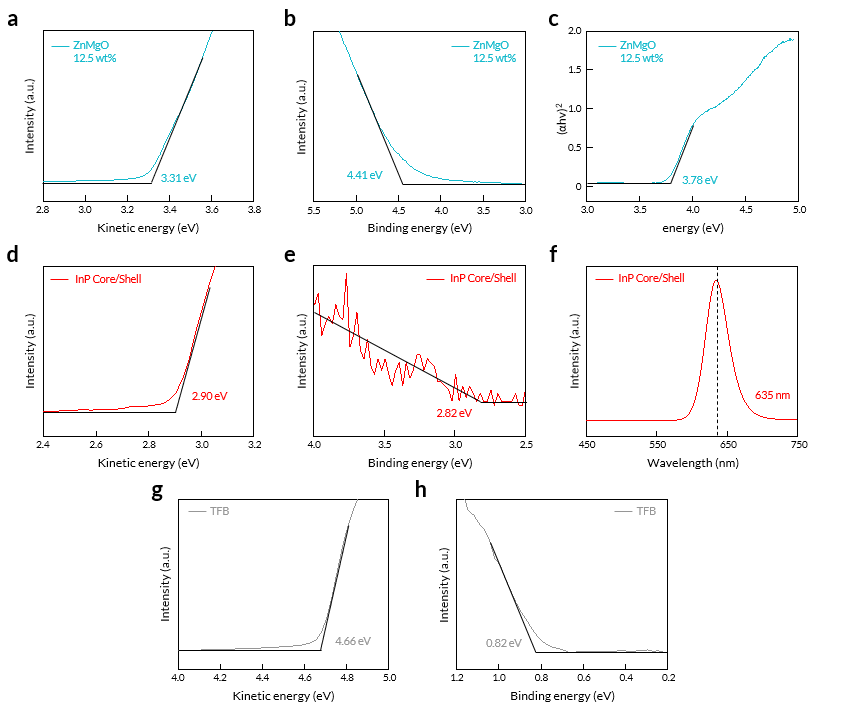
 Supplementary Fig.** **32 | Energy level characterization of materials used in rigid QLED.**

**a**, **b**, UPS spectra of ZnMgO (12.5 wt% Mg doped) film, showing (**a**) secondary cut-off and (**b**) valence band maximum (VBM). **c**, Tauc plot of ZnMgO (12.5 wt% Mg doped) film. **d**, **e**, UPS spectra of InP core/shell QD film, showing (**d**) secondary cut-off and (**e**) VBM. **f**, EL spectrum of rigid InP-based QLED. **g**, **h**, UPS spectra of TFB film. Bandgap of InP-based QD was calculated from the EL peak of QLED and the bandgap of TFB was obtained from Lumtec.

**
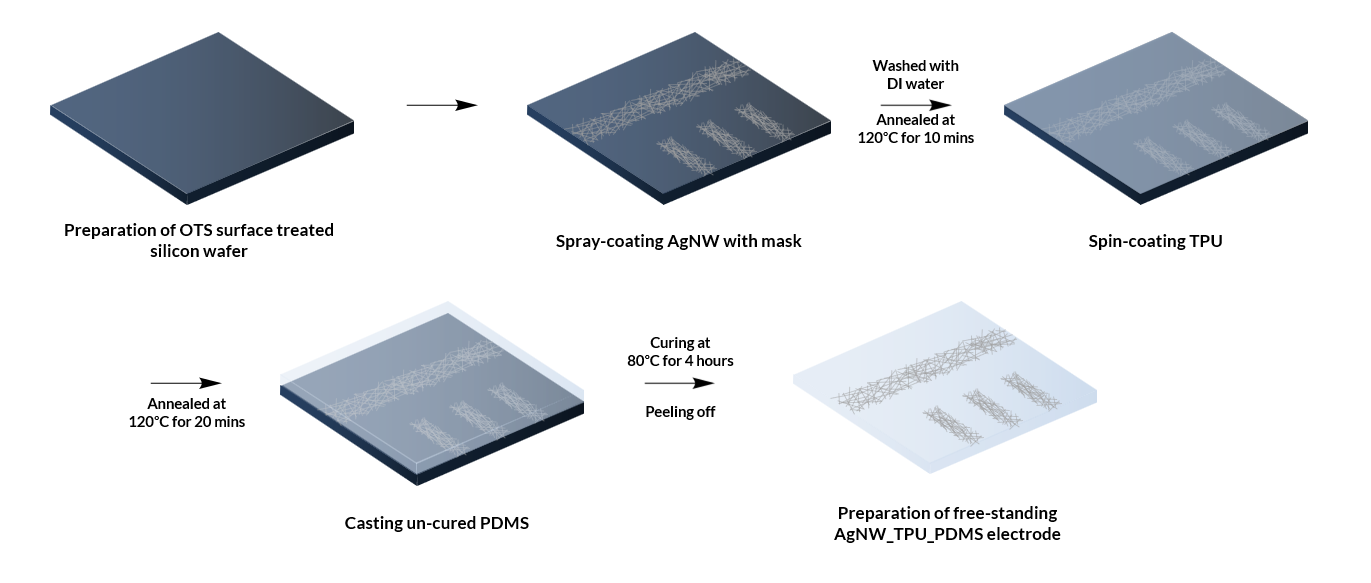
Supplementary Fig.** **33 | Schematic illustration of the fabrication procedure of stretchable AgNW electrode.**


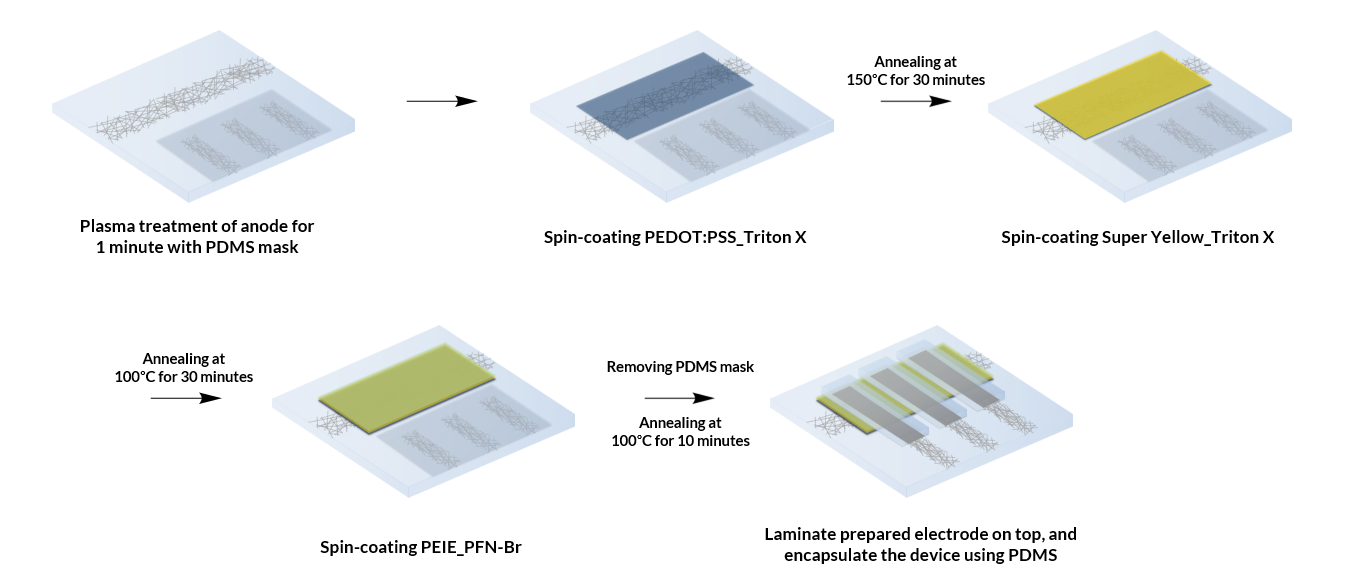
 **Supplementary Fig. 34 | Schematic illustration of fabrication procedure of SY-based intrinsically stretchable OLEDs.**


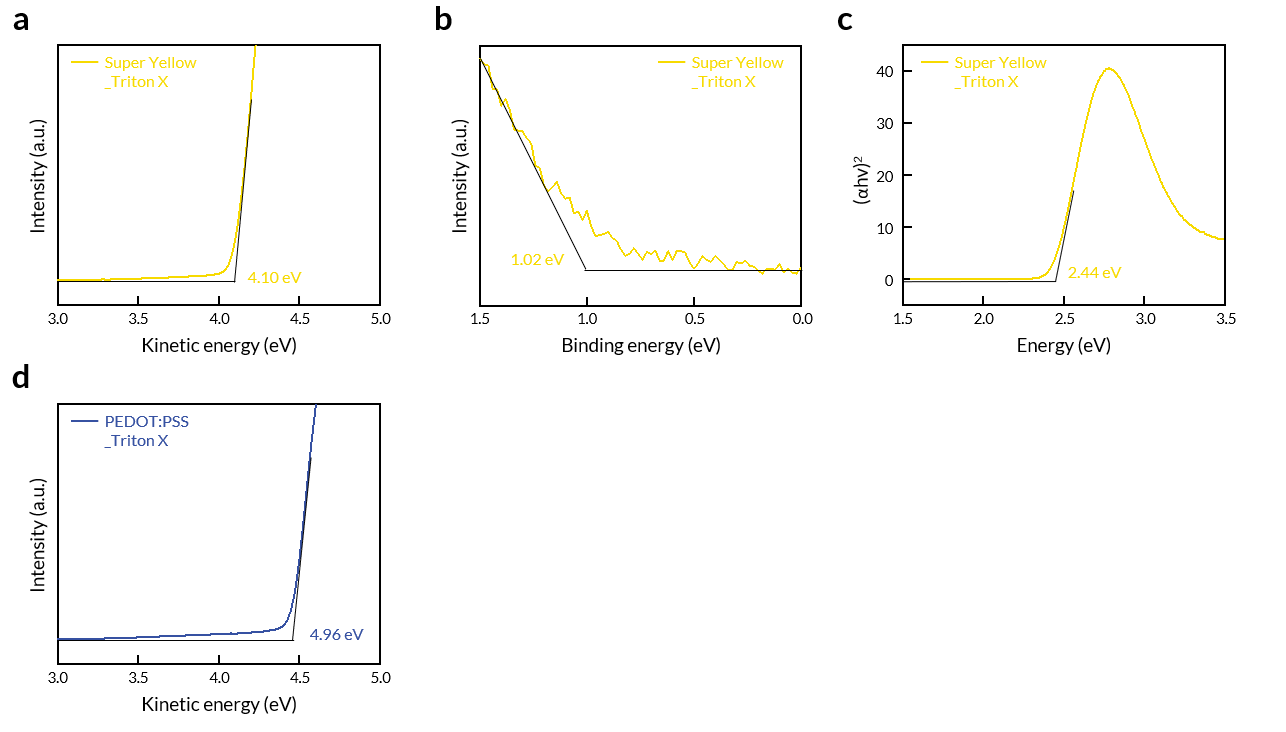


Supplementary Fig. 35 | Energy level characterization of materials used in SY-based intrinsically stretchable OLED.

**a**, **b**, UPS spectra of SY_Triton film coated on ITO substrate, showing (**a**) secondary cut-off and (**b**) HOMO edge. **c**, Tauc plot of SY_Triton film. **d**, UPS spectrum of PEDOT:PSS_Triton X coated on ITO substrate, showing its secondary cut-off.

**
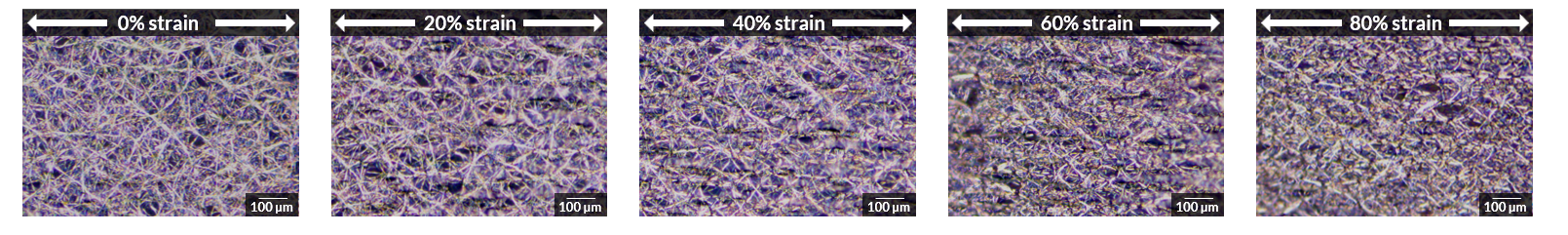
**

**Supplementary Fig. 36 | Stretchability of AgNW_PDMS_PU anode.**

Optical microscope images of AgNW_PDMS_PU film with varying uniaxial strain from 0% to 80%. The stretching direction is horizontal.

**
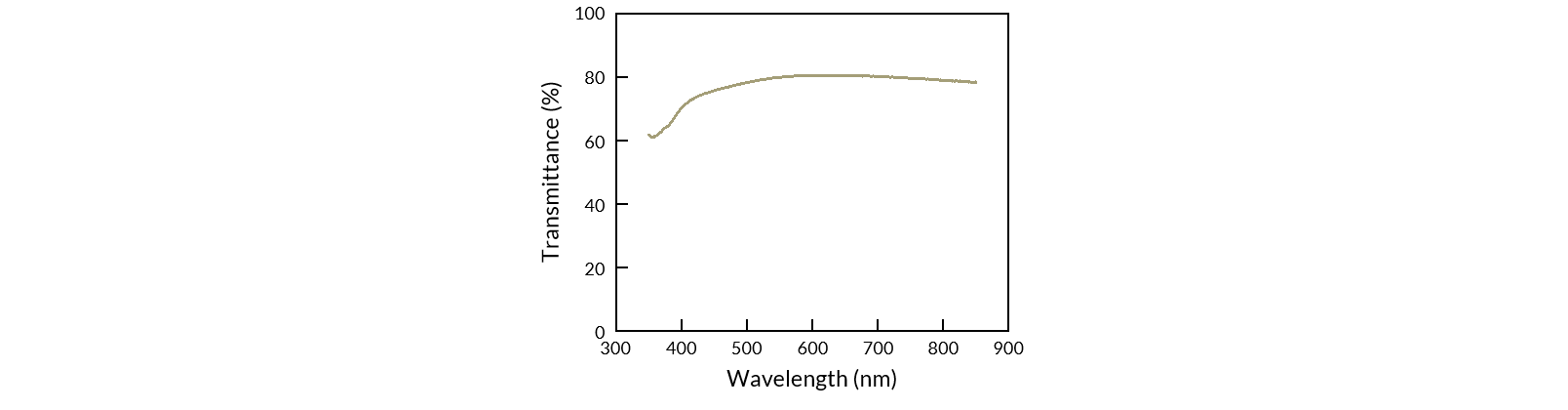
**

**Supplementary Fig. 37 | Transmittance of AgNW_PDMS_PU anode.**

Transmittance of AgNW_PDMS_PU anode as a function of wavelength from 350nm to 850nm.

**
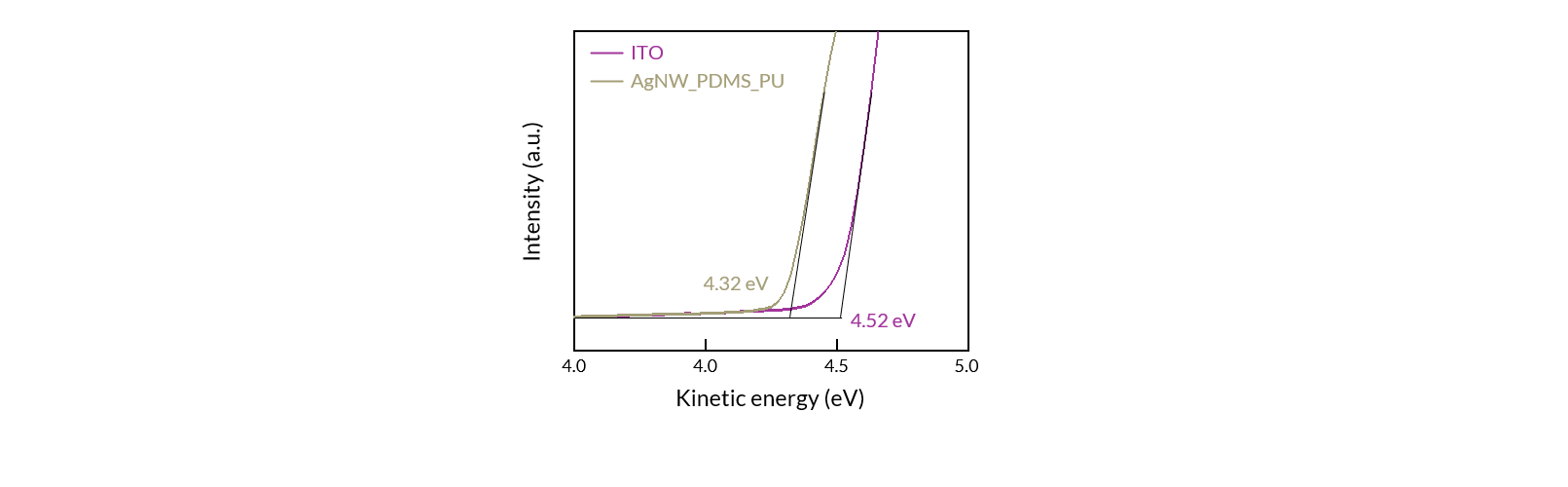
**

**Supplementary Fig. 38 | UPS spectra of ITO and AgNW_PDMS_PU.**


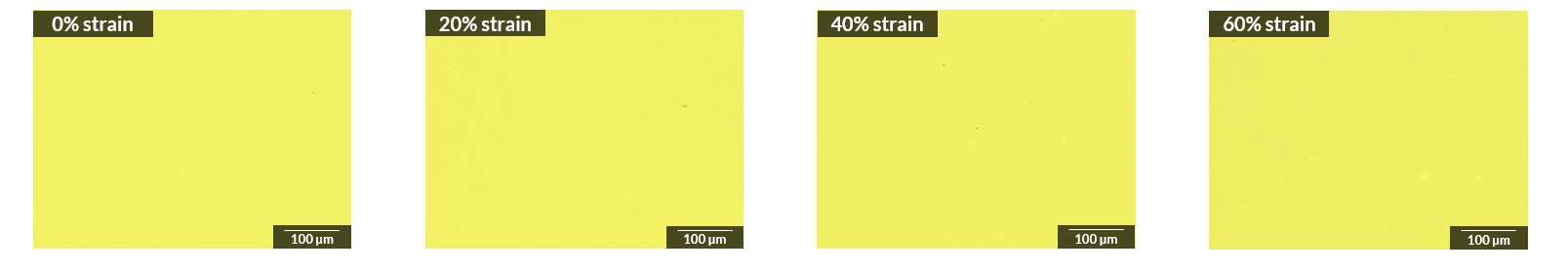
 Supplementary Fig. 39 | Stretchability of SY_Triton X film.

Optical microscope images of SY_Triton X film with varying uniaxial strain from 0% to 60%. No cracks were observed up to 60% strain. The stretching direction is horizontal.

**
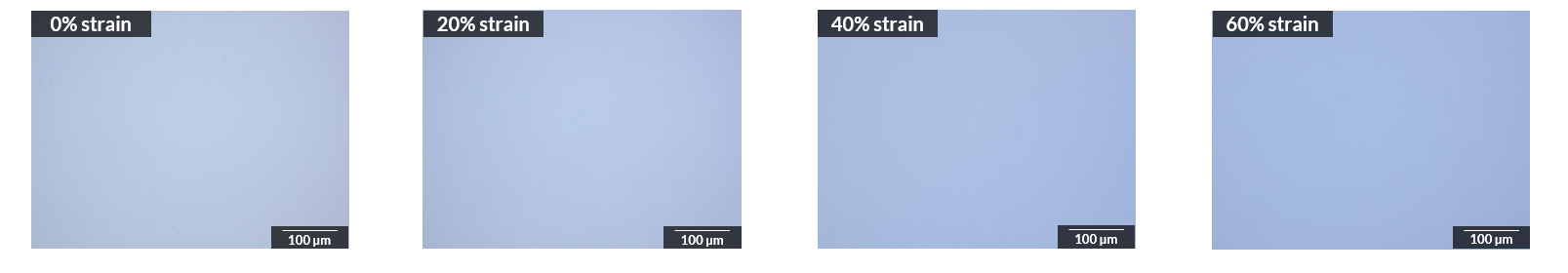
 Supplementary Fig.** **40 | Stretchability of PEDOT:PSS_Triton X.**

Optical microscope images of AI4083_Triton X film with varying uniaxial strain from 0% to 60%. No cracks were observed up to 60% strain. The stretching direction is horizontal.


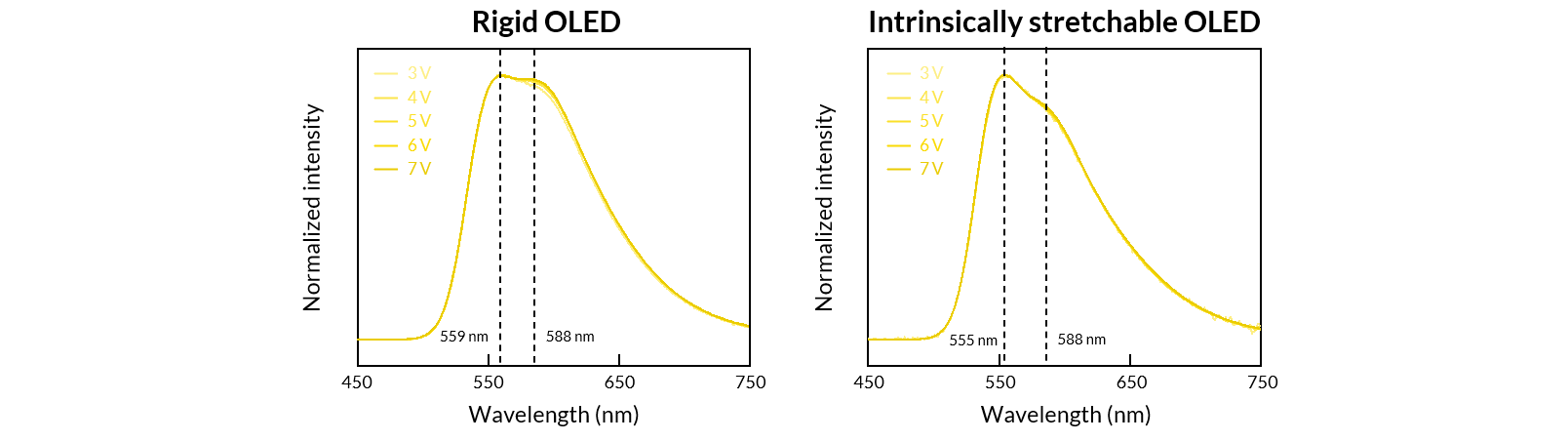


Supplementary Fig. 41 | EL spectrum of SY-based rigid and intrinsically stretchable OLEDs.
Both the rigid and intrinsically stretchable OLEDs exhibited comparable EL spectra with negligible spectral shifts with increasing bias, confirming their high color stability.


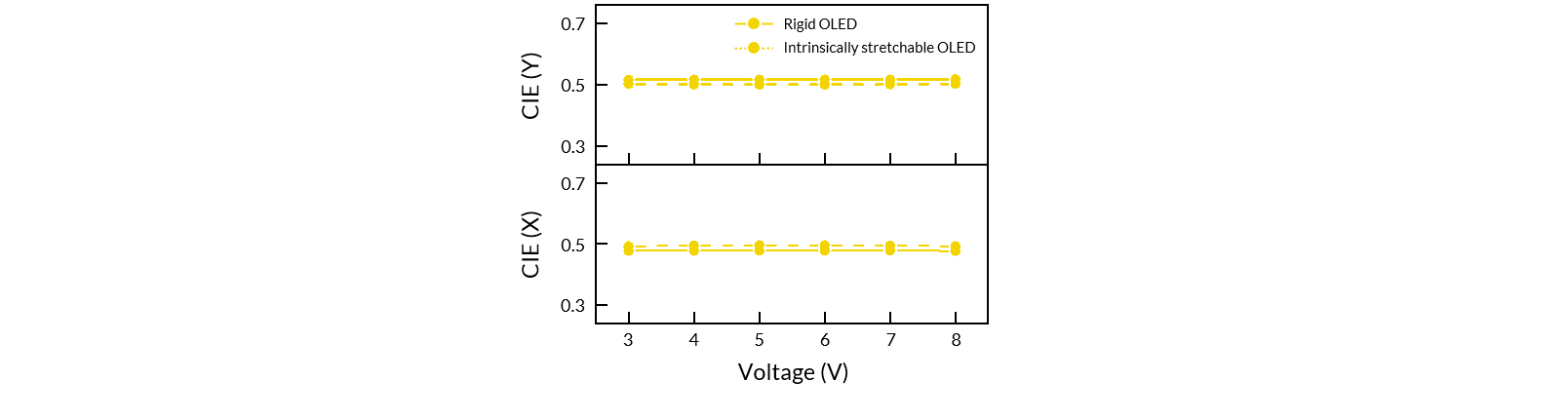


Supplementary Fig. 42 | CIE coordinates of SY-based rigid and intrinsically stretchable OLEDs.

Both the rigid and intrinsically stretchable OLEDs exhibited negligible changes in CIE coordinates with increasing bias, confirming their high color stability.


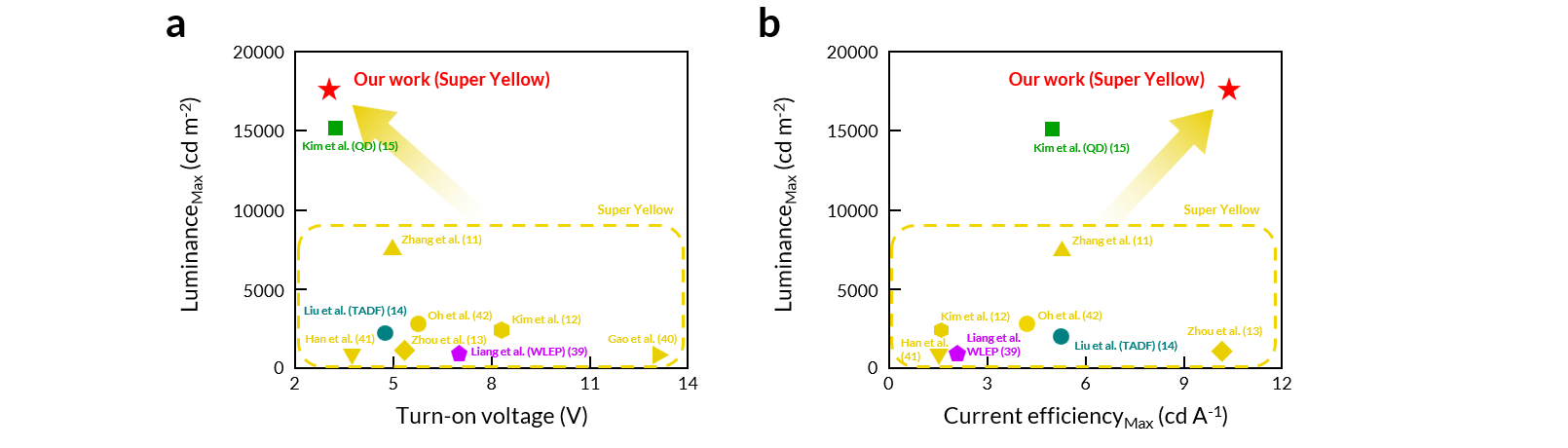


Supplementary Fig. 43 | Comparison of Hyb-LM-based intrinsically stretchable OLED with previously reported intrinsically stretchable LEDs (with LED characteristics measured from a single side).

**a**, Maximum luminance versus turn-on voltage. **b**, Maximum luminance versus maximum current efficiency. We note that luminance and current efficiency values were divided by two when the original reports provided the sum from both the anode and cathode sides. As shown in panels **a** and **b**, our device exhibits the highest luminance, the highest current efficiency, and the lowest turn-on voltage among the reported intrinsically stretchable LEDs. QD: Quantum Dot, TADF: Thermally Activated Delayed Fluorescence, WLEP: White Light-Emitting Polymer.


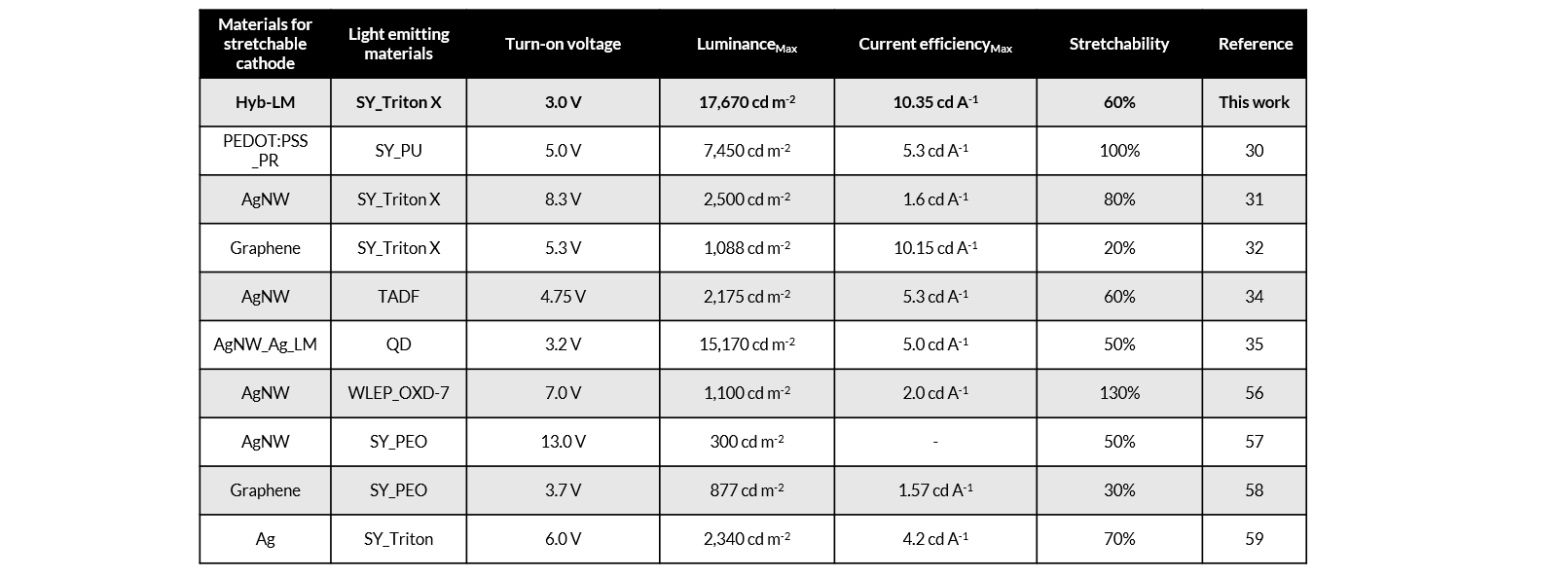


Supplementary Table 2 | Comparison of Hyb-LM-based intrinsically stretchable OLED with previously reported intrinsically stretchable LEDs (with LED characteristics measured from a single side).

^1^WLEP: White Light-Emitting Polymer


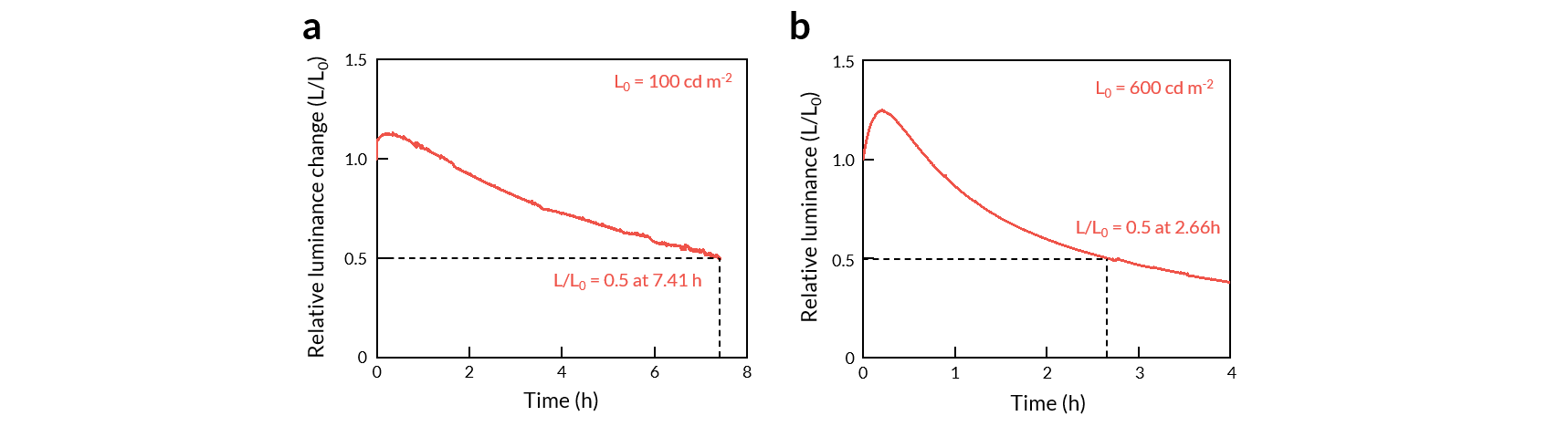


Supplementary Fig. 44 | Operational lifetime of the intrinsically stretchable OLED with Hyb-LM.

**a**, **b**, Device lifetime of intrinsically stretchable OLED with Hyb-LM at (**a**) 100 cd m^-2^ and (**b**) 600 cd m^-2^. (**a**) At 100 cd m^-2^, the device demonstrated a half-lifetime of 7.41 hours. (**b**) When the luminance was increased to 600 cd m^-2^, the device demonstrated a half-lifetime of 2.66 hours.


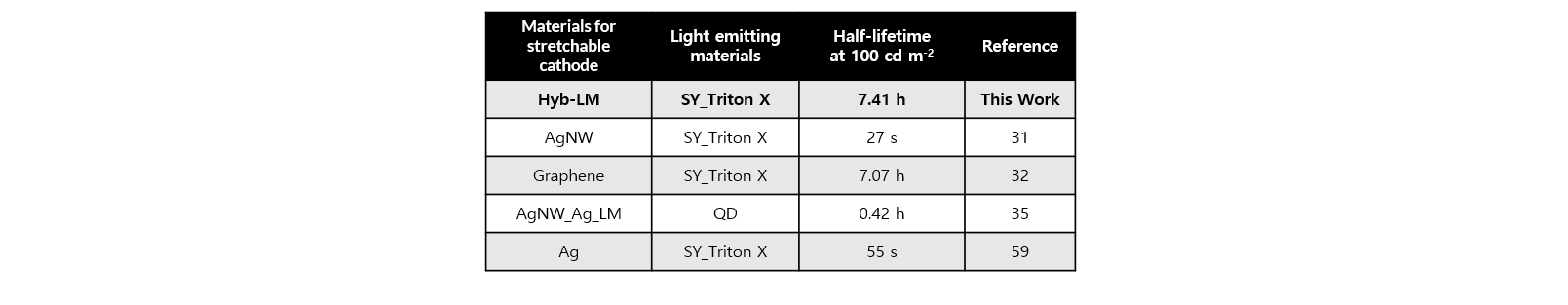


**Supplementary Table 3 | Comparison of operational lifetime of intrinsically stretchable OLED incorporating Hyb-LM with the previously reported intrinsically stretchable LEDs.**


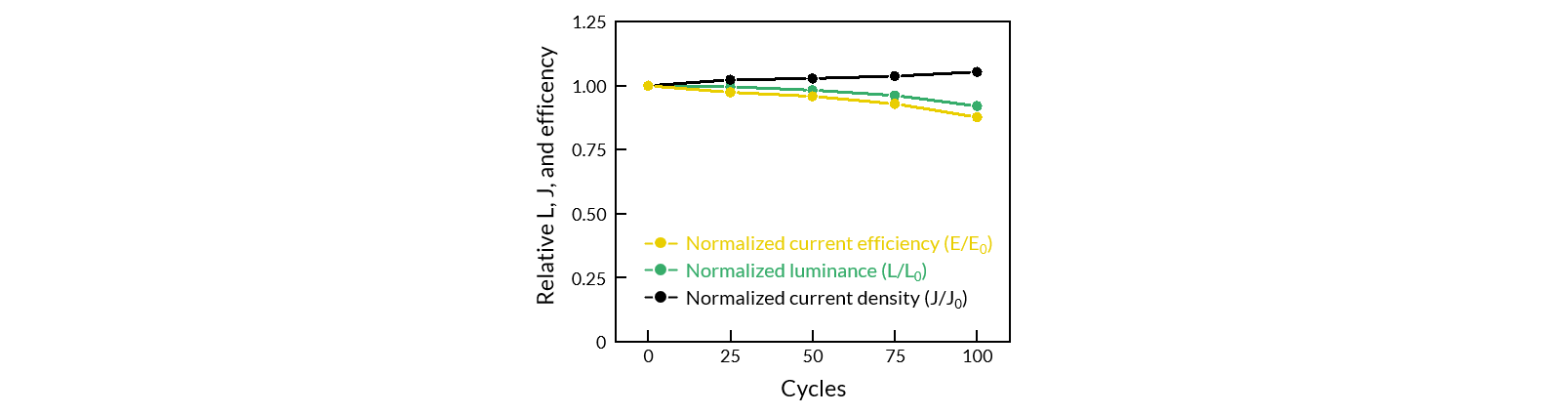


Supplementary Fig. 45 | Cyclic stability of intrinsically stretchable OLED with Hyb-LM.

Relative change of current efficiency of intrinsically stretchable OLED with Hyb-LM cathode under 100 cycles of 40% strain. The luminance, current density and current efficiency were 92%, 105% and 87% of their initial values. Degradation of intrinsically stretchable OLED with Hyb-LM is mainly attributed to the rupture of AgNWs under cyclic deformation.


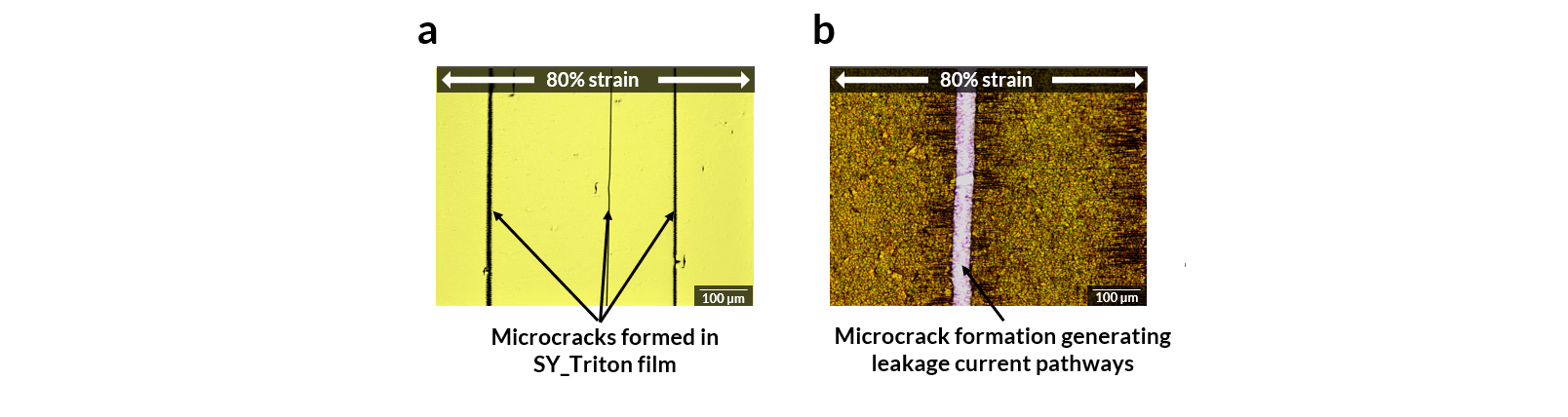


**Supplementary Fig. 46 |** **Microcrack formation in SY_Triton film under 80% uniaxial strain.**

**a,** optical microscope image of SY_Triton film under 80% uniaxial strain**. b**, Optical microscope image of the pixel of intrinsically stretchable OLED with Hyb-LM. When the device is stretched to 80%, microcracks form in the SY-Triton film, leading to generation of leakage current pathways between the Hyb-LM and AgNW electrodes. The generation of leakage current pathway is identified as the primary failure mechanism that limits the strain tolerance of the device.


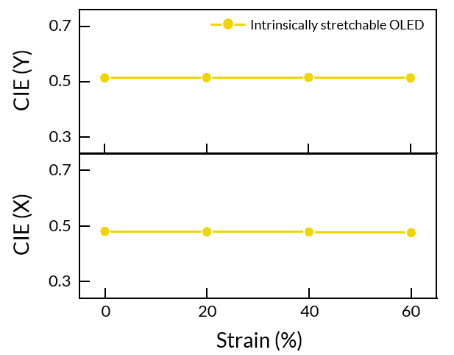


Supplementary Fig. 47 | CIE coordinates of intrinsically stretchable OLED under strain.

The intrinsically stretchable OLED showed stable CIE coordinates under strain, implying its excellent color stability under mechanical deformation.


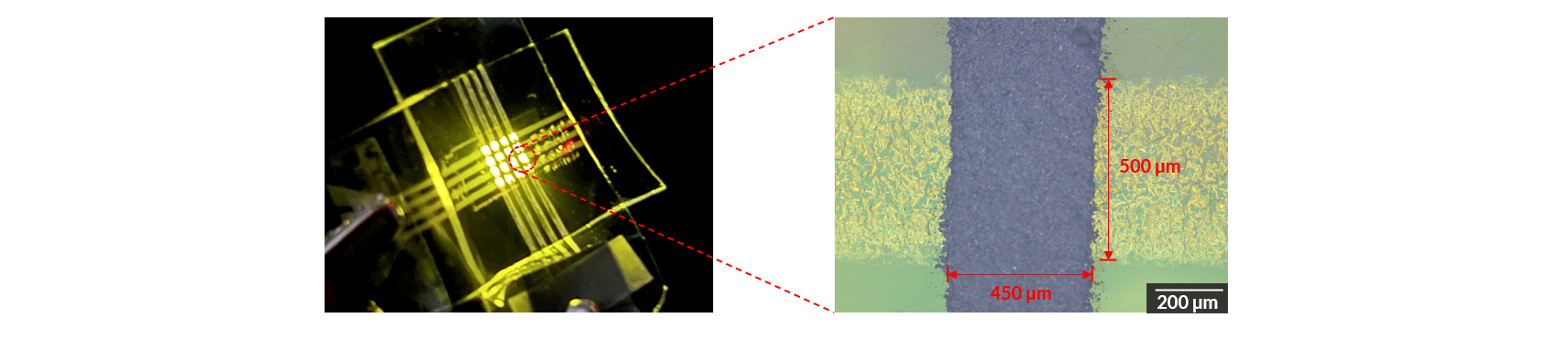
 Supplementary Fig. 48 | Optical microscope image of the pixel in crossbar structured intrinsically stretchable OLED.

Optical microscope image on the right shows a pixel with dimensions of 450 µm × 500 µm, which, to the best of our knowledge, is the smallest reported among crossbar-structured intrinsically stretchable LEDs.


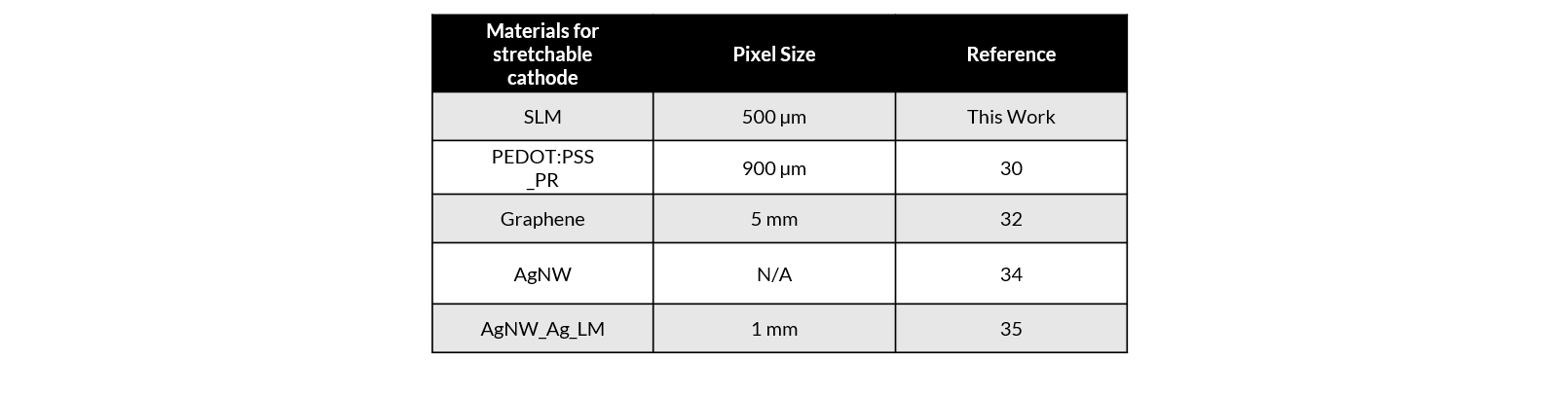


Supplementary Table. 4 | Comparison of the pixel size of the Hyb-LM-based intrinsically stretchable OLED with those of previously reported intrinsically stretchable LEDs.

**References**

1. S. Wang, J. Xu, W. Wang, G.-J. N. Wang, R. Rastak, F. Molina-lopez, J. W. Chung, S. Niu, V. R. Feig, J. Lopez, T. Lei, S.-K. Kwon, Y. Kim, A. M. Foudeh, A. Ehrlich, A. Gasperini, Y. Yun, B. Murmann, J. B.-H. Tok & Z. Bao, *Nature* **2018**, 555, 83.

2. A. Miyamoto, S. Lee, N. F. Cooray, S. Lee, M. Mori, N. Matsuhisa, H. Jin, L. Yoda, T. Yokota, A. Itoh, M. Sekino, H. Kawasaki, T. Ebihara, M. Amagai & T. Someya, *Nat. Nanotech.* **2017**, 12, 907.

3. D.-H. Kim, N. Lu, R. Ma, Y.-S. Kim, R.-H. Kim, S. Wang, J. Wu, S. M. Won, H. Tao, A. Islam, K. J. Yu, T. Kim, R. Chowdhury, M. Ying, L. Xu, M. Li H.-J. Chung, H. Keum, M. McCormick, P. Liu, Y.-W. Zhang, F. G. Omenetto, Y. Huang, T. Coleman, J. A. Rogers, *Science* **2011**, 333, 838.

4. Y.-Q. Zheng, Y. Liu, D. Zhong, S. Nikzad, S. Liu, Z. Yu, D. Liu, H.-C. Wu, C. Zhu, J. Li, H. Tran, J. B.-H. Tok, Z. Bao, *Science* **2021**, 373, 88.

5. Y. Dai, S. Wai, P. Li, N. Shan, Z. Cao, Y. Li, Y. Wang, Y. Liu, W. Liu, K. Tang, Y. Liu, M. Hua, S. Li, N. Li, S. Chatterji, H. C. Fry, S. Lee, C. Zhang, M. Weires, S. Sutyak, J. Shi, C Zhu, J. Xu, X. Gu, B. Tian, S. Wang, *Science* **2024**, 386, 431.

6. N. Li, Y. Li, Z. Cheng, Y. Liu, Y. Dai, S. Kang, S. Li, N. Shan, S. Wai, A. Ziaja, Y. Wang, J. Strzalka, W. Liu, C. Zhang, X. Gu, J. A. Hubbell, B. Tian, S. Wang, *Science* **2023**, 381, 686

7. Y. Li, N. Li, W. Liu, A. Prominski, S. Kang, Y. Dai, Y. Liu, H. Hu, S. Wai, S. Dai, Z. Cheng, Q. Su, P. Cheng, C. Wei, L. Jin, J. A. Hubbell, B. Tian & S. Wang, *Nat. Commun.* **2023**, 14, 4488.

8. X. Chen, P. Chen, J. Shi, Z. liu, P. Zhang, Y. Wang, Y. Zhang, C. Yan, L. Yang, B. Su, *Adv. Funct. Mater.* **2025**, 35, 2425794.

9. Z. Li, S. Zhang, Q. Wang, Y. Xu, Y. Li, X. Chen, P. Chen, D. Chen, Y. Shi, B. Su, *Adv. Mater.* **2024**, 36, 2409142.

10. X. Chen, F. Manshaii, D. Tang, Y. Xu, Z. Li, M. Chen, P. Chen, Y. Li, S. Zhang, L. Yang, J. Chen, B. Su, *Adv. Sci.* **2025**, 12, 2505157.

11. W. Lee, H. Kim, I. Kang, H. Park J. Jung, H. Lee, H. Park, J. S. Park, J. M. Yuk, S. Ryu, J.-W. Jeong, J. Kang. *Science* **2022**, 378, 637.

12. J. Liang, L. Li, X. Niu, Z. Yu & Q. Pei, *Nat. Photonics* **2013**, 7, 817.

13. T. Sekitani, H. Nakajima, H. Maeda, T. Fukushima, T. Aida, K. Hata & T. Someya, *Nat. Mater.* **2009**, 8, 494.

14. Y. Wang, Y. Liu, Y. Guo, *FlexMat* **2025**, 2, 312.

15. H. Uoyama, K. Goushi, K. Shizu, H. Nomura & C. Adachi, *Nature* **2012**, 492, 234.

16. Y.-H. Won, O. Cho, T. Kim, D.-Y. Chung, T. Kim, H. Chung, H. Jang, J. Lee, D. Kim & E. Jang. *Nature* **2019**, 575, 634.

17. J. S. Kim, J.-M. Heo, G.-S. Park, S.-J. Woo, C. Cho, H. J. Yun, D.-H. Kim J. Park, S.-C. Lee, S.-H. Park, E. Yoon, N. C. Greenham & T.-W. Lee, *Nature* **2022**, 611, 688.

18. M. Du, Y. Chen, M. Mai, T. Fan, Q. jin, Y. Zhang, L. Duan, *FlexMat* **2024**, 1, 46.

19. H. Li, G. Chen, Z. Ye, K. Feng, J. Huang, G. Xie, Y. Tao, *FlexMat* **2024**, 1, 173.

20. X.-C. Li, H. Sun, Z. Wang, W. Yang, Q. Wang, C. Wu, J. Chen, Q. jiang, L.-J. He, Q. Xue, W. Huang & W.-Y. Lai, *Nat. Commun.* **2025**, 16, 3321.

21. J.-W. Jeong, J. G. McCall, G. Shin, Y. Zhang, R. Al-Hasani, M. Kim, S. Li, J. Y. Sim, K.-I. Jang, Y. Shi, D. Y. Hong, Y. Liu, G. P. Schmitz, L. Xia, Z. He, P. Gamble, W. Z. Ray, Y. Huang, M. R. Bruchas, J. A. Rogers, *Cell* **2015**, 162, 662.

22. D. Kim, T. Yokota, T. Suzuki, S. Lee, T. Woo, W. Yukita, M. Koizumi, Y. Tachibana, H. Yawo, H. Onodera, M. Sekino, T. Someya, *Proc. Natl Acad. Sci. USA* **2020**, 117, 21138.

23. S. Choi, Y. Na, J. Lee & K. C. Choi, *Proc. Int. Conf. Display Technol. (ICDT)* **2020**, 52, 279.

24. H. Yu, J. Kim, H. Kim, N. Barange, X. Jiang, F. So, *ACS Appl. Mater. Interfaces* **2020**, 12, 36409.

25. R.-H. Kim, D.-H. Kim, J. Xiao, B. H. Kim, S.-I. Park, B. Panilatis, R. Ghaffari, J. Yao, M. Li, Z. Liu, V. Malyarchuk, D. G. Kim, A.-P. Le, R. G. Nuzzo, D. L. Kaplan, F. G. Omenetto, Y. Huang, Z. Kang & J. A. Rogers, *Nat. Mater.* **2010**, 9, 929.

26. Y. Shi, B. Zhang, J. Zhao, J. Qin, K. Bai, J. Yu, X. Zhang, *FlexMat* **2024**, 1, 150.

27. M. S. White, M. Kaltenbrunner, E. D. Głowacki, K. Gutnichenko, G. Kettlgruber, I. Graz, S. Aazou, C. Ulbricht, D. A. M. Egbe, M. C. Miron, Z. Major, M. C. Scharber, T. Sekitani, T. Someya, S. Bauer & N. S. Sariciftci, *Nat. Photonics* **2013**, 7, 811.

28. D. Yin, J. Feng, R. Ma, Y.-F. Liu, Y.-L. Zhang, X.-L. Zhang, Y.-G. Bi, Q.-D. Chen & H.-B. Sun, *Nat. Commun.* **2016**, 7, 11573.

29. S. Jeong, H. Yoon, B. Lee, S. Lee, Y. Hong, *Adv. Mater. Technol.* **2020**, 5, 2000231.

30. Z. Zhang, W. Wang, Y. Jiang, Y.-X. Wang, Y. Wu, J.-C. Lai, S. Niu, C. Xu, C.-C. Shih, C. Wang, H. Yan, L. Galuska, N. Prine, H.-C. Wu, D. Zhong, G. Chen, N. Matsuhisa, Y. Zheng, Z. Yu, Y. Wang, R. Dauskardt, X. Gu, J. B.-H. Tok & Z. Bao, *Nature* **2022**, 603, 624.

31. J.-H. Kim and J. W. Park, *Sci. Adv.* **2021**, 7, eabd9715.

32. H. Zhou, S. J. Han, A. K. Harit, D. H. Kim, D. Y. Kim, Y. S. Choi, H. Kwon, K.-N. Kim, G.-T. Go, H. J. Yun, B. H. Hong, M. C. Suh, S. Y. Ryu, H. Y. Woo, T.-W. Lee, *Adv. Mater.* **2022**, 34, 2203040.

33. X.-C. Li, L. Yao, W. Song, F. Liu, Q. Wang, J. Chen, Q. Xue, W.-Y. Lai, *Angew. Chem., Int. Ed.* **2022**, 62, e202213749.

34. W. Liu, C. Zhang, R. Alessandri, B. T. Diroll, Y. Li, H. Liang, X. Fan, K. Wang, H. Cho, Y. Liu, Y. Dai, Q. Su, N. Li, S. Li, S. Wai, Q. Li, S. Shao, L. Wang, J. Xu, X. Zhang, D. V. Talapin, J. J. de Pablo & S. Wang, *Nat. Mater.* **2023**, 22, 737.

35. D. C. Kim, H. Seung, J. Yoo, J. Kim, H. H. Song, J. S. Kim, Y. Kim, K. Lee, C. Choi, D. Jung, C. Park, H. Heo, J. Yang, T. Hyeon, M. K. Choi & D.-H. Kim, *Nat. Electron.* **2024**, **7**, 365.

36. Z. Yu, X. Niu, Z. Liu, Q. Pei, *Adv. Mater.* **2011**, 23, 3989.

37. R. C. Chiechi, E. A. Weiss, M. D. Dickey, G. M. Whitesides, *Angew. Chem. Int. Ed.* **2008**, 47, 142.

38. J. Zhang, Q. Lu, Y. Li, T. Li, M.-H. Lu, Y.-F. Chen, and D. Kong, *ACS Mater. Lett.* **2021**, 1104.

39. E. J. Markvicka, M. D. Bartlett, X. Huang, C. Majidi, *Nat. Mater.* **2018**, 17, 618.

40. S. Lee, S. A. Jaseem, N. Atar, M. Wang, J. Y. Kim, M. Zare, S. Kim, M. D. Bartlett, J.-W. Jeong, and M. D. Dickey, *Chem. Rev.* **2025**, 125, 3551 (2025).

41. L. Sun, J. Wang, H. Matsui, S. Lee, W. Wang, S. Guo, H. Chen, K. Fang, Y. Ito, D. Inoue, D. Hashizume, K. Mori, M. Takakuwa, S. Lee, Y. Zhou, T. Yokota, K, Fukuda, and T. Someya, *Sci. Adv.* **2024**, 10, eadk9460.

42. J. Noh, G.-U. Kim, S. Han, S. J. Oh, Y. Jeon, D. Jeong, S. W. Kim, T.-S. Kim, B. J. Kim, and J.-Y. Lee, *ACS Energy Lett.* **2021**, 6, 2512.

43. J. Wang, Y. Ochiai, N. Wu, K. Adachi, D. Inoue, D. Hashizume, D. Kong, N. Matsuhisa, T. Yokota, Q. Wu, W. Ma, L. Sun, S. Xiong, B. Du, W. Wang, C.-J. Shih, K. Tajima, T. Aida, K. Fukuda & T. Someya, *Nat. Commun*. **2024**, 15, 4902.

44. M. D. Dickey, R. C. Chiechi, R. J. Larsen, E. A. Weiss, D. A. Weitz, G. M. Whitesides, *Adv. Funct. Mater.* **2008**, 18, 1097.

45. S. Lee, S. Oh, S. Han, D. Lee, J. Lee, Y. Kim, H.-Y. Jeong, J.-W. Lee, M.-H. Lee, W. B. Ying, S. Jeong, S. Lee, J. Kim, Y. H. Kim, B. J. Kim, E. Jeon, T.-S. Kim, S. Cho and J.-Y. Lee, *Energy Environ. Sci.* **2024**, 17, 8915.

46. G. Park, G.-H. Lee, W. Lee, J. Kang, S. Park, S. Park, *Adv. Funct. Mater.* **2024**, 34, 2309660.

47. S. Zhu, J.-H. So, R. Mays, S. Desai, W. R. Barnes, B. Pourdeyhimi, M. D. Dickey, *Adv. Funct. Mater.* **2013**, 23, 2308.

48. S. Veerapandian, W. Jang, J. B. Seol, H. Wang, M. Kong, K. Thiyagarajan, J. Kwak, G. Park, G. Lee, W. Suh, I. You, M. E. Kılıç, A. Giri, L. Beccai, A. Soon & U. Jeong, *Nat. Mater.* **2021**, 20, 533.

49. S. Liu, D. S. Shah & R. Kramer-Bottiglio, *Nat. Mater.* **2021**, 20, 851.

50. A. Fassler and C. Majidi, *Adv. Mater.* **2015**, 27, 1928.

51. G.-H. Lee, Y. R. Lee, H. Kim, D. A. Kwon, H. Kim, C. Yang, S. Q. Choi, S. Park, J.-W. Jeong & S. Park, *Nat. Commun.* **2022**, 13, 2643.

52. H. Seo, G.-H. Lee, J. Park, D.-Y. Kim, Y. Son, S. Kim, K. S. Nam, C. Yang, J. Won, J.-Y. Bae, H. Kim, S.-K. Kang, S. Park, J. Kang & S. Park, *Nat. Commun.* **2025**, 16, 4944.

53. Y. Zhou, C. Fuentes-Hernandez, J. Shim, J. Meyer, A. J. Giordano, H. Li, P. Winget, T. Papadopoulos, H. Cheun, J. Kim, M. Fenoll, A. Dindar, W. Haske, E. Najafabadi, T. M. Khan, H. Sojoudi, S. Barlow, S. Graham, J.-L. Bredas, S. R. Marder, A. Kahn and B. Kippelen, *Science* **2021**, 336, 327.

54. S. Ohisa, T. Kato, T. Takahashi, M. Suzuki, Y. Hayashi, T. Koganezawa, C. R. McNeill, T. Chiba, Y.-J. Pu, and J. Kido, *ACS Appl. Mater. Interfaces* **2018**, 10, 17318.

55. N. Matsuhisa, S. Niu, S. J. K. O’Neill, J. Kang, Y. Ochiai, T. Katsumata, H.-C. Wu, M. Ashizawa, G.-J. N. Wang, D. Zhong, X. Wang, X. Gong, R. Ning, H. Gong, I. You, Y. Zheng, Z. Zhang, J. B.-H. Tok, X. Chen & Z. Bao, *Nature* **2021**, 600, 246.

56. J. Liang, L. Li, K. Tong, Z. Ren, W. Hu, X. Niu, Y. Chen, and Q. Pei, *ACS Nano* **2014**, 8, 1590.

57. H. Gao, S. Chen, J. Liang, Q. Pei, *ACS. Appl. Mater. Interfaces* **2016**, 8, 32504.

58. S. J. Han, H. Zhou, H. Kwon, S.-J. Woo, T.-W. Lee, *Adv. Funct. Mater.* **2023**, 33, 2211150.

59. J.-H. Oh, K.-H. Jeon & J.-W. Park, *Npj Flex. Electron.* 2024, **8**, 43.
